# Monitoring CMJ-derived metrics and subjective recovery across a competitive microcycle in semi-professional women’s volleyball

**DOI:** 10.1371/journal.pone.0328664

**Published:** 2026-09-01

**Authors:** Arturo Franco-Andrés, Pablo Casado-Martínez, Manuel Conejero-Suárez, Jaime González-García

**Affiliations:** 1 Faculty of Health Sciences, Universidad Francisco de Vitoria, Madrid, Spain; 2 Faculty of Health Sciences - HM Hospitals, University Camilo José Cela, Madrid, Spain; SPRINT - Sport Physical Activity and Health Research & Innovation Center, PORTUGAL

## Abstract

**Purpose:**

Jumping ability is a crucial element for success in volleyball. The countermovement jump (CMJ) is a reliable, non-invasive, and quick tool to periodically assess neuromuscular fatigue and performance, which, in combination with subjective measures, can offer a comprehensive view of fatigue/recovery status. The study aimed to: a) analyze changes in CMJ after a women’s volleyball match based on playing time and b) evaluate the temporal perceived recovery and well-being. It was hypothesized that players would recover baseline values of CMJ and subjective variables 48 hours post-match.

**Methods:**

A descriptive, and repeated-measures design was proposed. Twelve volleyball players were evaluated for changes in CMJ before and after a match, and at 24, 48, and 72 hours post-match. Outcome variables (jump height and reactive strength index modified), kinetic variables (concentric impulse), and jump strategy variables (movement time and time to peak power) were analyzed. Perceived recovery and well-being scales assessed perceived recovery and well-being.

**Results:**

Significant changes were observed in jump height, concentric impulse, and reactive strength index modified between pre-match and 48 hours post-match in players with less playing time. In the high-play time group, significant changes in these variables were observed between pre-match and 48 and 72 hours. Perceived recovery and well-being were not significantly affected by match play.

**Conclusion:**

Competition did not significantly affect CMJ variables, suggesting that volleyball does not reduce jumping ability immediately after competing, which has direct implications for microcycle structure. The study highlights the importance of continuous monitoring of performance and recovery in volleyball. However, these findings should be interpreted with caution due to the small sample size of the study.

## Introduction

Jumping ability is a crucial element for success in volleyball. The main actions performed by players during the game largely depend on their jumping ability (e.g., spike, block or serve). Additionally, a relationship between jumping ability and the effectiveness of offensive actions has been demonstrated [1,2]. Similarly, differences in jumping ability among different levels of competition have been observed in both men and women [3,4].

To maximize performance, it is recommended to monitor athletes with the highest possible frequency, enabling the identification of relationships between load and injury risk [5]. This process involves the precise measurement and monitoring not only of the sports and non-sports loads that athletes face but also of their performance, emotional well-being, symptoms, and injuries [6]. The benefits of monitoring athletes are numerous: it may help us understand the cause of any changes in performance, increasing the understanding of training responses, detecting fatigue and recovery needs, as well as informing the planning and modification of training programs and competition schedules [5]. Additionally, it ensures the generation of appropriate load doses to minimize the risk of non-functional overtraining, injuries, and illnesses [7].

Beyond its established association with neuromuscular performance, countermovement jump (CMJ) outcomes, both in terms of final performance (e.g., jump height) and the underlying force-time characteristics (e.g., movement strategy, impulse generation, and phase-specific force application), can provide a sensitive estimation of the fatigue experienced by the athlete. In this context, the CMJ has been proposed as a potentially sensitive tool for detecting fatigue-induced changes and informing neuromuscular readiness for subsequent training sessions or competition [8]. This, in turn, may assist coaches and physical trainers in managing the training load in subsequent sessions [8,9]. Due to the aforementioned factors, of reliability, and the speed in collecting and analyzing data, the countermovement jump (CMJ) is widely used to assess athletes’ jumping ability and the effectiveness of different training programs [10]. Assessing neuromuscular performance has demonstrated excellent reliability both within and between days in volleyball players [9], making CMJ a comprehensive tool for frequent use in performance and fatigue evaluation processes in ecological contexts. With appropriate metric selection, CMJ analysis can provide valuable insights into athlete performance profiling, neuromuscular fatigue, and recovery status following injury [8]. In this context, incorporating alternative CMJ metrics beyond traditional ones represents an effective approach to fatigue monitoring, enabling coaches to make more informed training decisions and promote optimal neuromuscular recovery. However, given the multidisciplinary and holistic nature of fatigue etiology, using markers like those provided by CMJ testing alone does not provide a complete view of the process of training stimulus, fatigue, response, and adaptation. Therefore, it is advisable to include subjective assessments of well-being, in addition to monitoring training and competition loads and evaluating neuromuscular fatigue. These assessments provide complementary information to the neuromuscular fatigue markers derived from evaluations like the CMJ [11]. Total Quality Recovery Scale (TQR) [12] and Hooper Index (HI) [13], are two subjective evaluations of athlete well-being that complement neuromuscular fatigue analysis. The TQR evaluates from 1–10 the perceived recovery status of athletes using a visual analogic scale. It correlates with heart rate variability parameters in female volleyball players [14], and has also been shown as sensible to weekly training load variations in volleyball [15]. The Hooper Index (HI) and its sub-items (sleep quality, fatigue, stress, and muscle soreness) are promising tools for monitoring fatigue in team sports [16]. Each variable (sleep quality, fatigue, stress and muscle soreness) is assessed using a Likert-type scale ranging from 1 to 7, where lower scores indicate better status and higher scores reflect greater perceived strain, and the HI is subsequently calculated as the sum of these individual ratings. For example, the HI has shown an association with training load in professional football [17], with reduced values observed up to 72 hours post-match, supporting the use and combination of subjective assessments of recovery and form.

Despite the growing interest in monitoring fatigue and recovery in team sports, there is still limited evidence regarding the integrated analysis of objective neuromuscular measures and subjective recovery markers in team sports [18]. Furthermore, the influence of contextual factors such as match play time and subsequent training load on recovery dynamics remains poorly understood [19], particularly in female volleyball players. In addition, the reliability and practical applicability of different CMJ-derived metrics for fatigue monitoring in applied settings require further clarification. Therefore, there is a need for studies that adopt a holistic approach, integrating training load, subjective measures, and force–time derived variables, to better understand recovery profiles and support individualized decision-making in real-world performance environments. The primary objective of this study was to characterize temporal changes in CMJ-derived metrics and subjective recovery across a competitive microcycle in semi-professional women’s volleyball players according to playing time. Additionally, the study sought to examine the sensitivity of different CMJ variables to detect fatigue-related changes and to compare recovery responses according to match play time and subsequent training load.

## Methods

### Design

**Fig 1:**
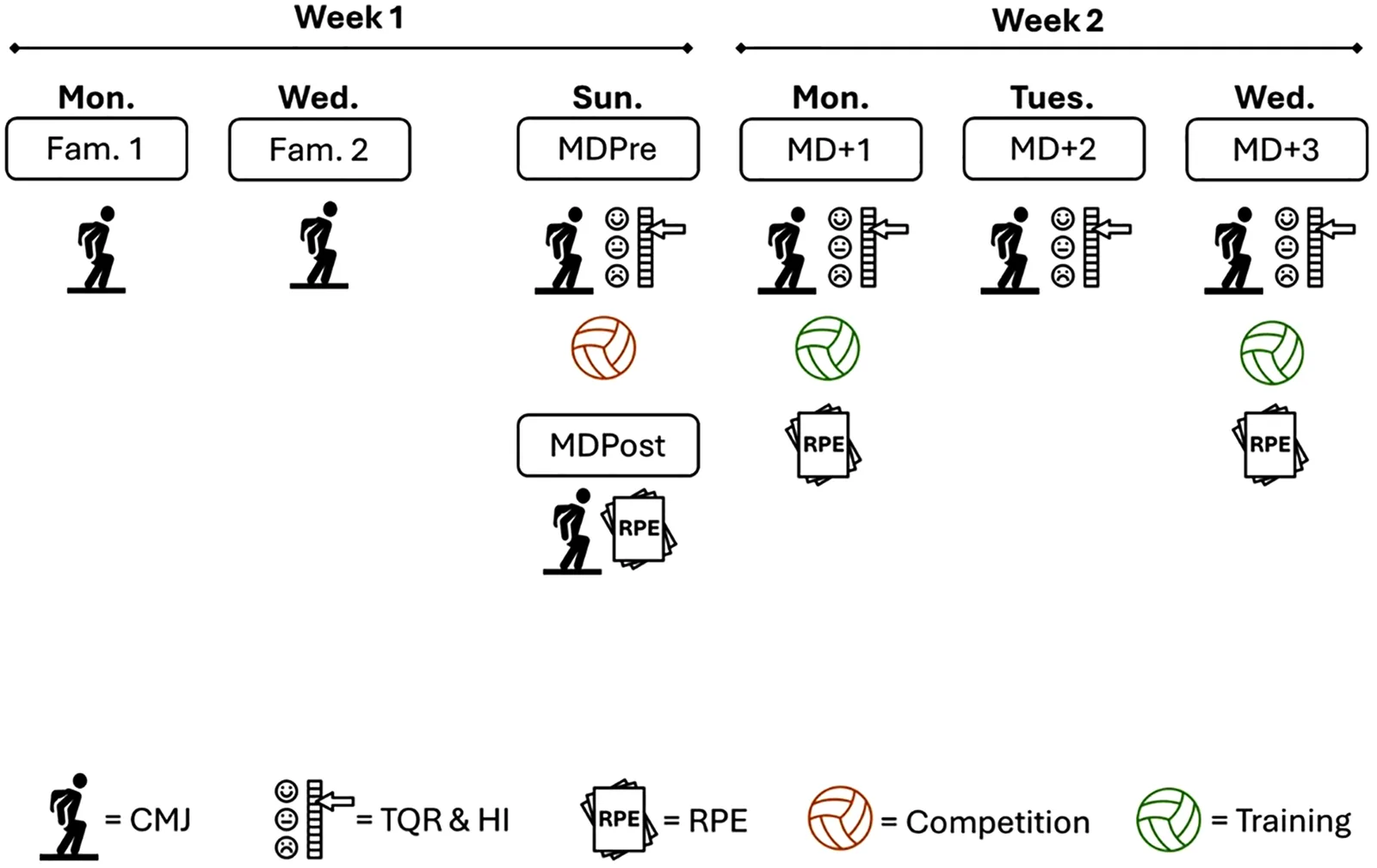
Study timeline across the competitive microcycle. Countermovement jump (CMJ) assessments were performed before the match (MDPre), immediately after the match (MDPost), and 24 h (MD + 1), 48 h (MD + 2), and 72 h (MD + 3) after the match. Total Quality Recovery (TQR) and Hooper Index (HI) questionnaires were completed on match day and on MD + 1, MD + 2, and MD + 3.

To address the research objectives, a descriptive, repeated measures, observational, multivariable, and prospective design was employed. During the first two sessions, participants were familiarized with the CMJ test, and the reliability and sensitivity of its metrics was established. The following five observations constituted the experimental period of the study. In Observation 1 (match day, MD), the CMJ was performed before the official warm-up. Observations 2, 3, 4 and 5 were conducted immediately post-match, at 24, 48, and 72 hours (Fig 1), respectively. All observations were carried out after the standardized warm-up, which is described in detail below. HI and TQR were measured in the morning of match day, MD + 1, MD + 2 and MD + 3.

### Participants

Twelve female volleyball players volunteered to participate in the study (22.8 ± 3.4 years; 168 ± 6 cm in height; 63.9 ± 4.7 kg in mass; 6 ± 3 years of competitive experience). They are competitors in the second division of the Madrid Volleyball Federation during the 2023/2024 season. To be included in the study, participants must have had at least 2 years of volleyball training experience, have not suffered any injuries in the past six months, and have signed an informed consent form. The study complies with the requirements of the Helsinki Declaration.

### Warm-up

Prior to each assessment, a standardized warm-up following the RAMP Model (Raise, Activate and Mobilize, Potentiate) was conducted. This warm-up included five minutes of jogging, five minutes of dynamic stretching, mobility exercises (for the shoulders and hips), and core activation, finishing with one set of five CMJs and one set of five CMJs with rebound. Rest intervals of two minutes were allowed between each warm-up block. To ensure optimal performance in the subsequent evaluations, the intensity of the warm-up progressively increased within each warm-up.

### CMJ evaluation

A dual force platform system (ForceDecks FD4000, ForceDecks, London, UK) was used to evaluate the CMJ. The platforms were zeroed before each trial. They recorded at a frequency of 1000 Hz. Participants performed three attempts, with 10–15 seconds of rest between each. They were instructed with the verbal command: “hands on hips, maximum speed, and maximum height.” Before the first jump, participants were asked to stand on the platforms and remain stationary for one second until the platforms completed the weight measurement. The start of the jump was marked when a reduction in ground reaction forces of 20 N was detected. Body weight was calculated by averaging the force recorded during a weighing phase of at least one second of quiet standing, and body mass was subsequently obtained by dividing this value by the acceleration due to gravity. The following metrics were analyzed [8]:

The variables analysed were grouped into three categories. Jump outcomes included jump height, calculated from force–time data using the impulse–momentum relationship, and the modified reactive strength index (RSImod), obtained by dividing jump height by movement time. Kinetic variables included concentric impulse, calculated as the product of force and time. Jump strategy variables included movement time, defined as the time elapsed between the start of the movement and the onset of the flight phase, as well as time to peak power.

### Training and competition quantification

To analyze the match demands, an arbitrary units measure was used, calculated from the equation Load = session RPE × session duration (min) [20]. This method has shown strong associations with heart rate data or global positioning systems (GPS) in team sports [20]. Rate of perceived exertion (RPE) was reported by participants ten minutes after the match or training session ended [20]. Participants were instructed to rate their overall perceived exertion for the entire session, considering both physical and psychological effort, using the standardized 1–10 scale provided, where 10 represents maximal exertion. The formula was used to verify that the training load during the MD + 1 session was consistent across all players. Descriptive data of training load is shown in Table 1.

**Table 1:** Training and competition loads according to play-time group.

Play-time group	TL MD (AU)	TL MD + 1 (AU)
High	238 ± 127	234 ± 186
Low	46 ± 60† p = 0.011	450 ± 269* p = 0.048

Note. TL = training load; † = significant with respect to high; * = significant with respect to MD.

### Total quality recovery and hooper index

To evaluate perceived recovery status, the Total Quality Recovery (TQR) scale was used, which employs a Likert scale from 1 to 10, with 10 indicating the highest possible perceived recovery. Participants were instructed to rate their overall recovery status, considering both physical and mental aspects, with higher scores reflecting better perceived recovery. For assessing well-being, the Hooper Index (HI) was utilized, which evaluates sleep quality, fatigue, muscle soreness, and stress on a scale from 1 to 7 each. Participants were instructed to rate each variable according to their perceived status over the previous 24 hours, where lower scores indicate better well-being and higher scores reflect greater perceived strain. The Hooper Index is calculated by summing the scores of the four questionnaire items. This value can range from 4 to 28, with a lower score indicating better well-being. Both assessments were administered via a Google Forms questionnaire sent out every morning (9:00–10:00 am). Participants were instructed to complete the questionnaires based on the previous 24 hours.

### Statistical analysis

For statistical analysis, Jamovi software (Jamovi) was used. The normality of the data distribution was assessed using the Shapiro-Wilk test. Intraclass correlation coefficients (3,1) (ICCs) [21] with 95% confidence intervals (95% CI) were calculated within attempts in the same observation and evaluated using the following criteria: poor reliability (<0.5), moderate reliability (0.50–0.75), good reliability (0.75–0.90), and excellent reliability (>0.90) [22]. The standard error of measurement (SEM) was calculated as the standard deviation multiplied by the square root of (1 − ICC), and the minimal detectable change (MDC) was subsequently derived as SEM × 1.96 × √2, representing the smallest change that exceeds measurement error at the 95% confidence level. Additionally, the coefficient of variation (CV) was calculated as the ratio of the standard deviation to the mean, expressed as a percentage, to assess relative variability. Grouping by playing time (high or low) was performed using median split analysis (high play-time group: > 40 min; low play-time group: ≤ 40 min). A two-way ANOVA was conducted to identify differences in recovery profiles between players with high (n = 7) and low play-time (n = 5). The Holm correction was applied for post hoc comparisons to reduce the risk of Type I errors associated with multiple testing. Effect sizes (ES) between conditions were calculated through Cohen’s d and classified as follows: ≤ 0.2 (trivial), ≥ 0.2–0.6 (small), ≥ 0.6–1.2 (moderate), ≥ 1.2–2.0 (large), and ≥2 (very large) [23]. Statistical significance was set at p < 0.05. Results are presented as mean ± standard deviation.

## Results

The variables jump height (ICC = 0.98 [0.91–0.99]; SEM = 0.66 cm; MDC95 = 1.83 cm; mean CV = 1.83%) and concentric impulse (ICC = 0.99 [0.94–1.00]; SEM = 2.05 N·s; MDC95 = 6.68 N·s; mean CV = 1.11%) showed good to excellent reliability, although jump height with wide confidence intervals. The RSImod (ICC = 0.78 [0.32–0.94]; SEM = 0.04 m·s^-1^; MDC95 = 0.11 m·s^-1^; mean CV = 6.72%) showed good reliability. In contrast, movement time (ICC = 0.45 [−0.23–0.84]; SEM = 63.41 ms; MDC95 = 175.76 ms; mean CV = 6.03%) and time to peak power (ICC = 0.46 [−0.24–0.84]; SEM = 0.06 s; MDC95 = 0.18 s; mean CV = 6.61%) exhibited poor reliability. Given these reliability outcomes, movement time and time to peak power were excluded from further inferential analyses, as their limited reproducibility reduces their capacity to detect true changes beyond measurement error, compromising their suitability for monitoring purposes in this context.

**Fig 2:**
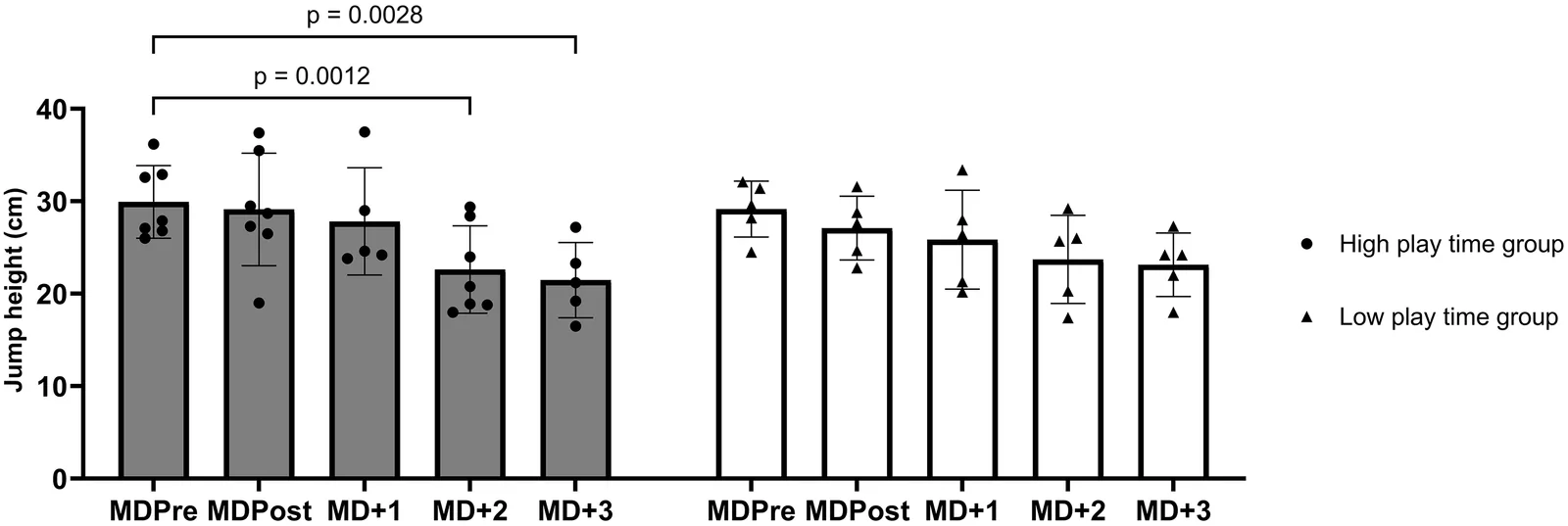
Jump height across the competitive microcycle according to match playing time. Bars represent mean values and error bars represent standard deviations; individual observations are superimposed. Circles and grey bars indicate the high play-time group (>40 min; n = 7), whereas triangles and white bars indicate the low play-time group (≤40 min; n = 5). Brackets and p values indicate significant within-group pairwise differences. MDPre, pre-match; MDPost, immediately post-match; MD + 1, 24 h post-match; MD + 2, 48 h post-match; MD + 3, 72 h post-match.

A main effect of time was observed for jump height [F(1.557, 14.01) = 7.006; p = 0.011; ηp² = 0.44] and concentric impulse [F(1.621, 14.59) = 11.11; p = 0.002; ηp² = 0.55] (Fig 2). No significant effect of group (based on playing time) or interaction effects were found for any variable.

**Fig 3:**
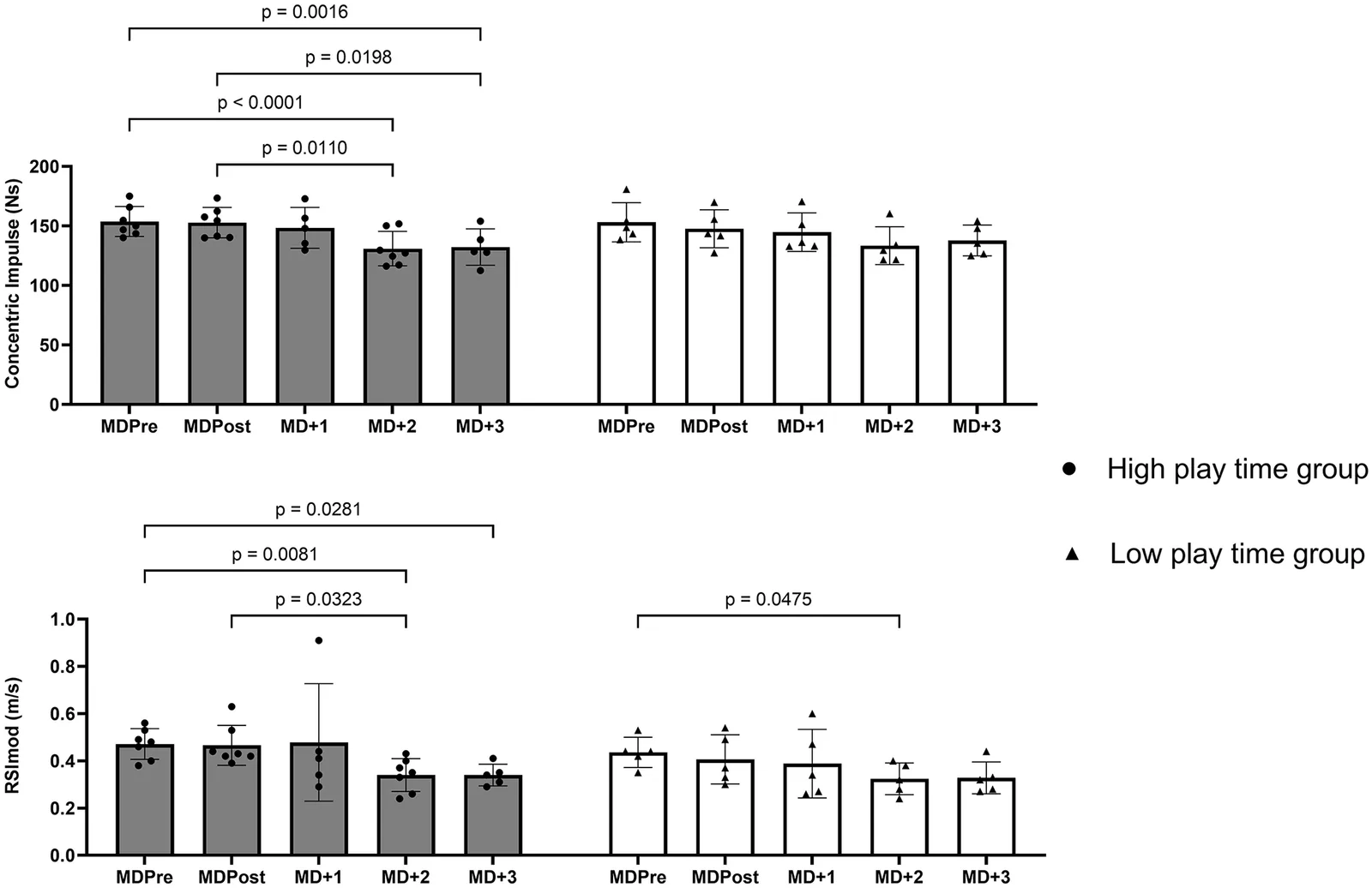
Concentric impulse (upper panel) and modified reactive strength index (RSImod; lower panel) across the competitive microcycle according to match playing time. Bars represent means and error bars represent standard deviations; individual observations are superimposed. Circles and grey bars indicate the high play-time group (>40 min; n = 7), whereas triangles and white bars indicate the low play-time group (≤40 min; n = 5). Brackets and p values indicate significant within-group pairwise differences. MDPre, pre-match; MDPost, immediately post-match; MD + 1, 24 h post-match; MD + 2, 48 h post-match; MD + 3, 72 h post-match.

In the high play-time group, significant reductions were observed at MD + 2 compared to MDPre in jump height, concentric impulse, and RSImod [jump height: *d* = −1.62 (−2.10 to −1.25); p = 0.001; concentric impulse: *d* = −1.57 (−1.78 to −1.37); p < 0.001; RSImod: *d* = −1.57 (−2.31 to −1.21); p = 0.008], and at MD + 3 compared to MDPre [jump height: *d* = −2.08 (−2.47 to −1.70); p = 0.003; concentric impulse: *d* = −1.61 (−1.87 to −1.36); p = 0.002; RSImod: *d* = −1.04 (−2.72 to −1.35); p = 0.028] (Fig 3). In the low play-time group, significant differences were observed only between MDPre and MD + 2 in RSImod (*d* = −0.60 [−2.67 to 1.48]; p = 0.047).

**Fig 4:**
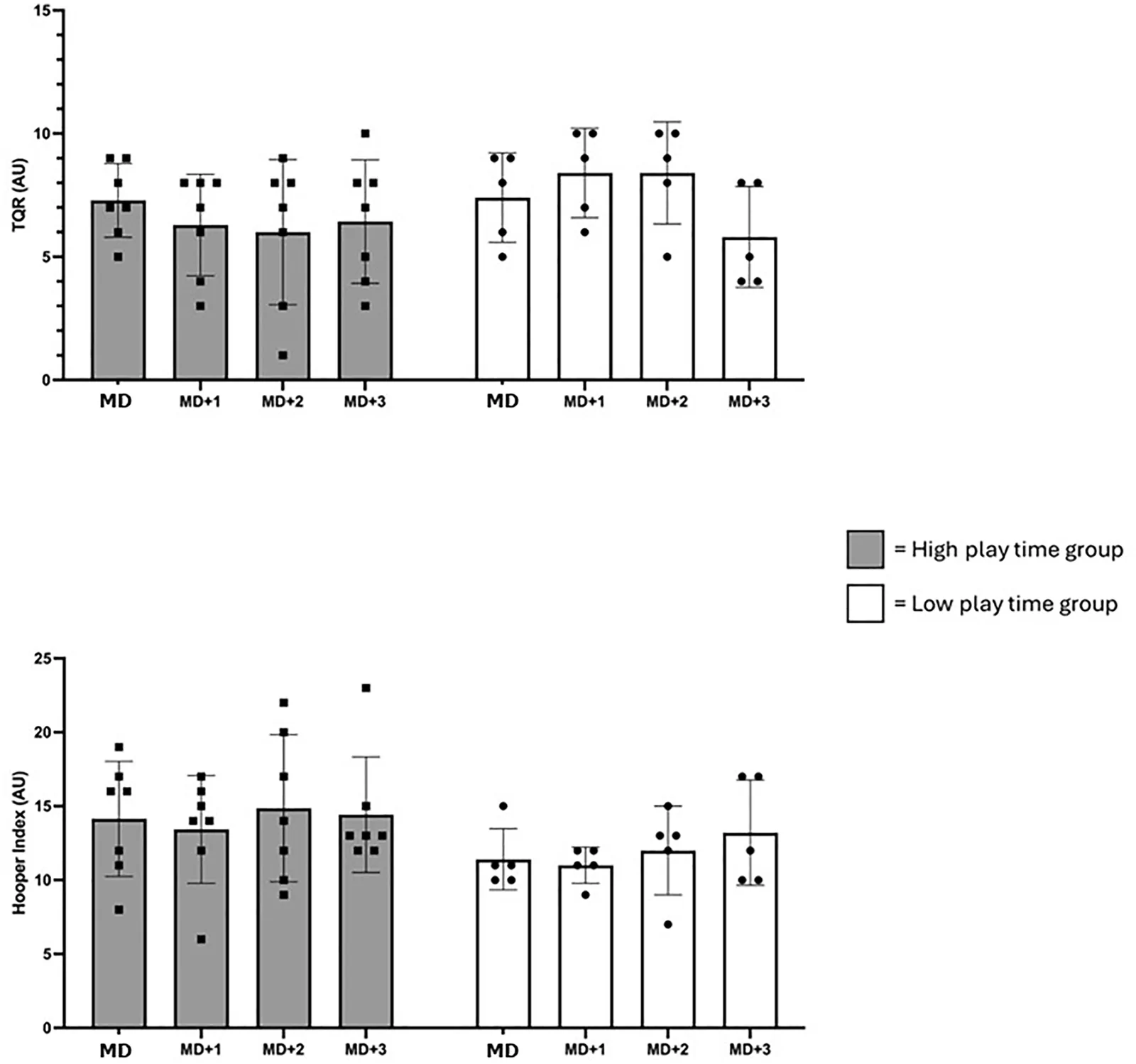
Total Quality Recovery (TQR; upper panel) and Hooper Index (HI; lower panel) across the competitive microcycle according to match playing time. Grey bars and squares represent the high play-time group (>40 min; n = 7), whereas white bars and circles represent the low play-time group (≤40 min; n = 5). Bars represent mean values, error bars represent standard deviations, and individual observations are superimposed. MD, match-day morning assessment; MD + 1, 24 h post-match; MD + 2, 48 h post-match; MD + 3, 72 h post-match. No significant time, group, or interaction effects were observed (p > 0.05).

For the subjective variables (TQR and HI), no significant differences were observed between match day and MD + 1, MD + 2, and MD + 3 in either group (p > 0.05; Fig 4).

## Discussion

The primary objective of this study was to characterize the recovery profile of various CMJ-derived metrics and perceived recovery across a competitive microcycle in semi-professional women’s volleyball players according to playing time. Our findings showed no evidence of immediate neuromuscular impairment following the match, as no significant changes were observed in jump outcomes (jump height and RSImod) or the force–time metric concentric impulse immediately post-match or 24 h later. However, significant reductions in jump height, concentric impulse, and RSImod emerged at MD + 2, whereas no significant changes were observed for TQR or HI at any assessment point. These findings suggest that neuromuscular alterations became more evident later in the competitive microcycle. Given the applied nature of the study, these temporal changes should be interpreted within the context of the cumulative neuromuscular stimulus experienced throughout the microcycle rather than as the isolated effect of competition. In particular, players were exposed not only to the competitive match but also to a scheduled MD + 1 training session, and the cumulative neuromuscular stimulus differed between subgroups because of their different exposure to match play and subsequent training. Therefore, differences in overall load exposure should be considered when interpreting the temporal changes observed in CMJ-derived variables.

The absence of immediate reductions in CMJ-derived variables following competition is consistent with previous findings in volleyball. Previous research has observed the same recovery pattern in professional male volleyball players [24], with no changes in jump height and movement time after two matches. Other studies aimed to measure changes in jump height throughout microcycles using inertial systems [25]. In these studies, no changes were observed in jump height, whether considering the mean of the attempts or the maximum value recorded in each observation, regardless of the microcycle session and time until the next competition. These findings suggest that no substantial immediate neuromuscular impairment was detected following competition. However, this should not be interpreted as evidence supporting high-load training on MD + 1, as delayed fatigue responses associated with accumulated neuromuscular loading may become evident during the subsequent 24–72 hours. The delayed reductions observed at MD + 2 are consistent with the cumulative neuromuscular demands imposed by both competition and the subsequent MD + 1 training session, highlighting the importance of interpreting these responses within the context of the competitive microcycle. In other team sports, competition has shown greater reductions in post-match jump capacity, kinetics, and kinematics. In fact, reductions of between 1.6 and 6 cm have been observed [26]. This can be explained by the differences in the physiological demands of the sports. In volleyball, the duration of each rally ranges from 3 to 40 seconds, followed by a brief recovery period averaging 12 seconds [27]. Additionally, the duration of the competition is typically shorter, although it depends on the progression of the match (66 minutes in the game analyzed). These differences in physiological demands (volume and density), combined with the high specialization of volleyball players in actions involving the stretch-shortening cycle [28], may explain the variation between volleyball and other sports in terms of reductions in jump height and the strategies employed by athletes, and consequently, in the fatigue generated by the competition.

Beyond the group-level analyses, the MDC approach provided additional insight into the sensitivity of the different CMJ-derived variables. Overall, concentric impulse consistently demonstrated the greatest ability to detect meaningful neuromuscular alterations throughout the competitive microcycle, whereas jump height and RSImod appeared less sensitive during the early stages of recovery. Specifically, only 17% and 44% of players exceeded the MDC for RSImod at MDPost and MD + 1, respectively, compared to 50% and 67% for concentric impulse, indicating a greater capacity of this variable to detect early fatigue-related alterations. Jump height showed moderate sensitivity at these time points, although changes were not consistently observed across all players, suggesting that performance outcomes may be partially preserved during the initial phase of recovery. At 48 h post-match (MD + 2), a clearer and more consistent fatigue response emerged. Concentric impulse demonstrated maximal sensitivity, with 100% of players exceeding the MDC, while jump height also showed a high detection rate (83%). In contrast, RSImod reached 75% of players exceeding the MDC, indicating improved but still comparatively lower sensitivity. This pattern suggests that neuromuscular fatigue becomes more detectable at later stages, particularly when assessed through force–time derived metrics such as concentric impulse. Experimental evidence also indicates that fatigue can alter the CMJ force–time profile even when changes in jump height are limited, supporting the examination of variables beyond the final performance outcome [29]. Importantly, these responses must be interpreted within the context of the loading structure of the microcycle. Players were exposed not only to match load (238 ± 127.86 AU in high play-time and 46.4 ± 60.02 AU in low play-time groups), but also to a subsequent MD + 1 training session (234 ± 186.62 AU and 450 ± 269.95 AU, respectively). Notably, the match load accumulated by players with higher playing time represented only ~52–53% of the training load performed on MD + 1 by players with lower playing time, indicating that the overall stimulus imposed during the microcycle differed substantially between subgroups. Therefore, the observed fatigue responses are likely influenced not only by competition, but also by the subsequent training stimulus, reinforcing the need to interpret neuromuscular changes within the broader context of total load exposure. This highlights a key limitation of aggregated analyses, as grouping players with substantially different loading profiles may obscure meaningful neuromuscular responses.

Although no significant interaction effects were observed, the MDC analysis suggested that players with lower playing time tended to exhibit a more consistent pattern of detectable neuromuscular alterations. In this subgroup, concentric impulse recorded maximal sensitivity, with 100% of players exceeding the MDC at both MD + 2 and MD + 3, while jump height reached similar detection levels only at later time points. In contrast, RSImod exhibited a delayed and less consistent response, with only 17% and 50% of players exceeding the MDC at MDPost and MD + 1, respectively, increasing to 83% and 75% at MD + 2 and MD + 3. These observations suggest that under conditions of a more homogeneous neuromuscular stimulus, the sensitivity of force–time metrics, particularly concentric impulse, may be enhanced, reinforcing its value for monitoring neuromuscular fatigue in applied settings. In line with these findings, jump height remained relatively stable until MD + 2, suggesting that neuromuscular performance was largely preserved during the first 24 h following competition. However, despite this preserved performance, the MD + 1 session was followed by reductions in both jump outcomes (jump height: d = −0.74, 95% CI [−1.52, 0.04]; RSImod: d = −0.45, 95% CI [−1.01, 0.10]) and kinetics (concentric impulse: d = −0.92, 95% CI [−1.57, −0.27]), while subjective measures (TQR: d = −0.28, 95% CI [−1.08, 0.51]; HI: d = 0.27, 95% CI [−0.37, 0.90]) recorded only small changes. This partially contrasts with previous work [8], suggesting that fatigue is primarily reflected through alterations in jump strategy. In the present study, the absence of detectable changes in strategy variables may be explained by their limited reliability, as well as by the specific neuromuscular demands of volleyball. The high specificity of ballistic training adaptations, characterized by rapid contractions and efficient motor unit recruitment, together with the relatively long intrinsic recovery periods within the sport, may allow athletes to maintain rapid force production capabilities despite underlying fatigue. Consequently, neuromuscular impairments may be more readily detected through force–time derived metrics such as concentric impulse rather than through performance outcomes or temporal strategy variables [30].

Collectively, these findings are consistent with observations reported in other team sports. Following competition in both male [31] and female soccer [32], and after a training volume similar to that of the MD + 1 session (90 minutes), reductions have been observed in the capacity to develop maximum power in a CMJ, jump height, and other physiological variables, which may not fully recover until after 48 hours and could require up to 72 hours to return to baseline levels [31]. In the case of rugby, the same phenomenon has been observed [26]. Our findings are consistent with the hypothesis that greater cumulative neuromuscular loading may be associated with longer recovery periods. Given all this information, it is essential to monitor the post-training response according to the training load to identify the recovery periods. This approach helps to minimize the risk of overtraining and maximizes neuromuscular adaptations to the training program [6]. These findings reinforce the importance of considering cumulative neuromuscular load when scheduling subsequent training sessions.

Based on previously observed recovery periods in team sports, it has been suggested that the session following a match should focus on recovery and involve a reduced training volume [25]. Within the present competitive context, where the scheduled MD + 1 training session imposed a greater neuromuscular stimulus than the match itself, it may not be essential to prescribe a recovery-focused session immediately after competition. This is because athletes have already regained their neuromuscular capacity without affecting TQR and HI values, which remained unchanged across the post-match assessments. The stability of response in these measures suggests that neuromuscular recovery may not be fully reflected in perceptual (TQR) or well-being measures (HI), highlighting the multidimensional and potentially dissociated nature of recovery processes. In this context, the integration of objective neuromuscular measures such as CMJ performance with subjective assessments (TQR and HI) may provide a more comprehensive and ecologically valid framework for monitoring athlete fatigue and recovery. While perceptual measures are easy to implement and capture important psychophysiological dimensions [33], they may be influenced by external and contextual factors (e.g., mood, stress, sleep, or cognitive load), potentially limiting their sensitivity to detect subtle neuromuscular impairments. In contrast, CMJ-derived variables, particularly outcome measures such as jump height and force–time metrics, offer an objective assessment of neuromuscular status, potentially enabling the identification of fatigue profiles that may not be reflected in subjective reports. However, these interpretations should be considered with caution, as the present findings are based on a relatively small sample and a specific applied context. Therefore, while the combined use of subjective and objective monitoring approaches appears promising, its practical application should be interpreted as context-dependent, and further research with larger samples is needed to confirm these observations and support more generalized recommendations.

Identifying individual recovery periods requires a holistic monitoring approach that integrates training load (TL), subjective measures (wellness and TQR), and objective neuromuscular assessments such as CMJ-derived variables. While TL provides information about the external stimulus imposed on the athlete, wellness and TQR capture the perceived psychophysiological response, and CMJ metrics offer an objective evaluation of neuromuscular status. The combined interpretation of these domains allows practitioners to better understand the relationship between load, fatigue, and recovery, facilitating more precise and individualized training prescriptions. This approach may enable practitioners to individualise training prescription according to the athlete’s neuromuscular status and cumulative load exposure [34,35]. However, given that recovery responses are multifactorial and influenced by contextual factors beyond training and competition load, reliance on a single monitoring tool may lead to incomplete interpretations [18]. Therefore, integrating subjective and objective indicators within an ecologically valid framework appears essential to optimize decision-making and microcycle structuring, particularly in applied settings such as volleyball.

This study presents several limitations that should be acknowledged. First, the relatively small sample size limits the generalizability of the findings. Second, the absence of a control group prevents causal inferences and limits the ability to compare the observed outcomes against a standardized reference. However, it is not ecologically or ethically valid in this applied setting to include a control condition. Third, data were collected from a single match, which restricts the representativeness of the results and may not capture variability across different competitive contexts or time points. In addition to this point, training load was not directly controlled or quantified in the present study, and this may have influenced our ability to fully interpret the neuromuscular performance and recovery responses across the observation period. An important consideration is that the observed changes cannot be attributed exclusively to match play, as players also completed a scheduled training session on MD + 1 and individual recovery-related behaviours outside the team environment (e.g., stretching, additional physical activity, or other recovery strategies) were not controlled. Consequently, the present findings should be interpreted as the neuromuscular responses occurring throughout a real competitive microcycle rather than as isolated effects of competition or recovery. Finally, player position was not considered due to our limited sample size; however, this factor in future investigations may highlight potential differences in match demands and recovery profiles between roles. Overall, these findings should be interpreted with caution, as they are exploratory in nature and are intended to provide preliminary insights rather than definitive conclusions regarding post-match recovery dynamics.

## Conclusion

No substantial immediate impairments were detected within the first 24 h following a semi-professional volleyball match. However, neuromuscular fatigue became more evident at 48 hours, likely reflecting the combined effect of match and subsequent training load, particularly in players with lower playing time. Concentric impulse emerged as the most sensitive variable to detect meaningful changes beyond measurement error, supporting the use of force–time metrics for monitoring neuromuscular fatigue. In contrast, subjective measures (TQR and well-being) did not reflect these changes, highlighting the need for a holistic monitoring approach. From a practical perspective, high-intensity training on MD + 1 may be feasible when recovery is adequate, but training decisions should be individualized by integrating training load, subjective responses, and CMJ-derived metrics.
