# Supplementary material for: Monitoring CMJ-derived metrics and subjective recovery across a competitive microcycle in semi-professional women’s volleyball
Source: PLoS One. 2026 Sep 1;21(9):e0328664. doi: 10.1371/journal.pone.0328664 (PMC13533353; doi:10.1371/journal.pone.0328664)
Supplement: Supplementary file 2 [file pone.0328664.s004.pdf]

This manuscript investigates the temporal patterns of neuromuscular fatigue and subjective recovery in semi-professional female volleyball players across a competitive microcycle. The authors employed countermovement jump (CMJ) metrics and subjective assessments—specifically the Total Quality Recovery (TQR) scale and the Hooper Index (HI)—to evaluate twelve athletes at multiple time points: pre-match, immediately post-match, and at 24, 48, and 72 hours following competition.

The primary findings indicate that competition did not induce immediate neuromuscular impairment; however, significant declines in jumping performance, characterized by reductions in jump height, concentric impulse, and the modified reactive strength index (RSImod), emerged 48 hours post-match. The authors interpret this delayed response as the cumulative effect of both match-induced fatigue and the subsequent training loads performed within the microcycle. Notably, subjective recovery measures remained relatively stable and did not significantly mirror these objective neuromuscular changes, suggesting a potential dissociation between perceived and actual physical readiness.

Overall, this study provides practical insights into the application of force-time metrics in an ecological sporting environment. It concludes that concentric impulse is a particularly sensitive indicator for monitoring fatigue and emphasizes the necessity of a holistic monitoring approach to individualize training prescriptions and optimize recovery in women's volleyball.

Overall Evaluation

Overall, the authors have successfully addressed the previous reviewers' comments, and the manuscript has become substantially more robust. However, I believe the following minor points should be addressed to further improve the quality of the final manuscript.

Specific Comments

1. Consistency in Decimal Places (L122, Table 1, etc.)

Inconsistencies in the number of decimal places were observed in several locations, such as L122 and Table 1. Please ensure that these are standardized throughout the manuscript, taking into account the appropriate number of significant figures for each metric.

2. Clarification of Jump Height ICC Confidence Interval (L194)

In L194, is the reported 95% CI for the Jump height ICC (0.01–0.99) indeed correct? Given that the ICC is 0.98, this interval appears unusually wide. Please double-check this value.

3. Reporting Effect Sizes for ANOVA (L204–205)

For the ANOVA results presented in L204–205, it is recommended to include the effect size (e.g., partial eta squared, η_p^2) to provide a more complete statistical picture.

4. Wording for Significant Differences (L209)

In L209, since you are describing a statistically significant difference, the word "significant" should be added before "reductions" (e.g., "...significant reductions were observed...") to ensure clarity.

5. Notation of Effect Sizes in the Results Section (L210, etc.)

In the Results section (e.g., L210), it is unconventional to use the generic abbreviation "ES." Since you have calculated Cohen’s d, please use "d = XX" or "Cohen’s d = XX" for standard reporting.

6. Clarification of "each" (L252)

In L252, the term "each" in the phrase "the duration of each point" is somewhat ambiguous. Please clarify what this refers to (e.g., "each rally" or "each game point") to improve reader understanding.

7. Consistent Use of Abbreviations (L263)

In L263, since the full term "minimal detectable change" has already been defined earlier in the manuscript, you should use the abbreviation "MDC" only.

8. Spacing and Formatting (L308, etc.)

There are several instances of extra or missing spaces throughout the manuscript, including L308. Please conduct a thorough review to ensure consistent formatting.

9. Enhancement of Figure Captions (L505–506)

The captions for Figures 1 and 2 (L505–506) are currently too brief, making it difficult for the reader to interpret the data without referring back to the text. Please provide more descriptive and detailed captions for these figures.
