# Supplementary material for: Monitoring CMJ-derived metrics and subjective recovery across a competitive microcycle in semi-professional women’s volleyball
Source: PLoS One. 2026 Sep 1;21(9):e0328664. doi: 10.1371/journal.pone.0328664 (PMC13533353; doi:10.1371/journal.pone.0328664)
Supplement: Supplementary file 3 [file pone.0328664.s002.docx]

**Response to Reviewers**

**Manuscript title:** Monitoring jumping ability and subjective recovery in women's volleyball: acute effects of match play on countermovement jump and well-being
**Manuscript ID:** PONE-D-25-34659

**Dear Editor and Reviewers,**

We sincerely thank you for your careful evaluation of our manuscript and for the constructive and insightful comments provided. We greatly appreciate the time and expertise dedicated to this review process.

The comments have been extremely valuable and have allowed us to substantially improve the manuscript in terms of clarity, methodological transparency, and interpretative rigor. In particular, the revised version addresses the concerns raised by improving the structure and readability of the text, clarifying the rationale for the selected variables, refining the methodological descriptions, and adopting a more cautious and precise interpretation of the findings.

Below, we provide a point-by-point response to all comments provided buy reviewers in the decision letter.

Reviewer #1

**Response: We sincerely thank the reviewer for the careful and constructive evaluation of our manuscript. We fully agree that the original version required substantial revision in terms of clarity, structure, and interpretative caution.**

**In response to these comments, we comprehensively revised the manuscript. First, we reduced repetition and rewrote several sections of the Introduction and Discussion to improve readability, precision, and accessibility.**

**Second, we selected these metrics because they have been consistently described in the scientific literature as relevant indicators for assessing neuromuscular fatigue and monitoring performance-related changes in athletes. However, we acknowledge the reviewer’s important point regarding the interpretation of concentric impulse. Accordingly, we revised the Discussion to recognize that concentric impulse may reflect both force-related and time-related components, and we now interpret this variable more cautiously.**

**Regarding the time-related variables, we fully agree that the original interpretation was too strong given their poor reliability values. To address this concern, we added a specific paragraph in the Results section stating that movement time and time to peak power will not be further interpreted due to their low reproducibility and limited capacity to detect true changes during CMJ monitoring. Accordingly, these variables have been removed from the subsequent interpretation of the findings, and the Discussion, practical implications, and Conclusions have been revised to avoid drawing inferences from these outcomes.**

**Finally, we clarified the source of the data used for the reliability analyses and expanded the methodological explanation of these calculations. We also incorporated a more cautious discussion of the variability observed across post-match time points and its possible influence on reliability and sensitivity to detect meaningful effects.**

**We are grateful for the reviewer’s detailed annotated comments, which were instrumental in improving the manuscript substantially.**

Reviewer #2

Comment:
Summary: Biomechanical variables involved in jump mechanics of semi-professional level female volleyball players were evaluated before, immediately after one day after, two days after, and three days after match play.
Comments: The authors have a good premise for assessing female volleyball players. How does the neuromuscular system recovery after a match in regards to counter-movement jump performance? There are a number of limitations which the authors acknowledge for this study. The largest limitation is the grouping of players into high match-load versus lower match load. With an N = 12, this significantly reduces the statistical power. The authors also did not make it clear in the text of the document how many players were in each group. It is also not clear how the training cycle influenced these results. The authors discuss the cycles of training, but this was not mentioned as a mitigating factor in this study. Another aspect of the study which is not mentioned is the positioning of the players. Typically, in a volleyball match the front line players jump more frequently than the other players on the court. This was not a factor addressed by this manuscript. The lack of a control group and the number of participants makes it difficult to readily evaluate the overall meaning of these data.

**Response:
We sincerely thank the reviewer for the positive assessment of the study premise and for the thoughtful methodological comments.**

**We fully acknowledge that the small sample size is a major limitation, particularly when participants are subdivided into groups according to playing time. In the revised manuscript, we have made this limitation more explicit and have substantially moderated our interpretation of the subgroup analyses. We now clearly state that these findings should be interpreted as preliminary and context-specific rather than broadly generalizable. We have also clarified the number of participants included in each subgroup in the revised Methods/Results sections.**

**Regarding the influence of the training cycle, we agree that this factor required clearer treatment. We have revised the manuscript to explain more explicitly that the MD+1 training session likely influenced the post-match recovery profile and may have contributed to the changes observed at later time points, particularly at MD+2. We now discuss training load as an important contextual factor and interpret the findings accordingly.**

**We also agree that player position may influence match demands and jump exposure. Unfortunately, this variable was not included in the original study design and therefore could not be analyzed. We have now acknowledged this explicitly as an additional limitation in the revised Discussion, noting that positional role may affect the external and neuromuscular demands experienced during competition.**

**Finally, we agree that the absence of a control group and the limited number of participants restrict the strength of the conclusions. Accordingly, the revised manuscript places greater emphasis on these methodological limitations and avoids overly strong practical inferences. We are grateful for these comments, which have helped improve the transparency and overall balance of the manuscript. Nevertheless, despite these constraints, the study offers a high degree of ecological validity, as data were collected within a real-world, high-performance environment where experimental control is often not feasible. This context enhances the practical relevance of the findings, as they reflect authentic training and competition demands rather than artificially controlled conditions. Consequently, the results provide meaningful applied insights for practitioners working in similar elite settings, supporting evidence-informed decision-making while acknowledging the inherent methodological limitations.**

Specific comments

Comment (Methods):
How specific are the demographic information? It seems that if height was measured in cm, then a standard deviation of 6 cm will suffice and not 6.01 cm, likewise, the standard deviations for age and mass should be to one decimal point.

**Response: We thank the reviewer for this observation. The demographic data were revised to improve consistency and to avoid reporting an unnecessary degree of precision.**

Comment (Methods):
If N = 12 and there was a comparison between groups – and possibly uneven numbers between groups, the statistical power would seem to be lowered. Was a power analysis conducted prior to the initiation of the study? The authors do not provide the number of participants for each group.

**Response: We thank the reviewer for this important comment. No a priori power analysis was conducted prior to data collection. We acknowledge this as a limitation of the study and have now explicitly stated this in the revised manuscript. In addition, we have substantially moderated the interpretation of the subgroup analyses and inferential results accordingly. We also stated clearly in the methods section the number of participants in each group.**

Comment (Methods):
An explanation of the time periods of data collection are not clear. A pre-test was performed and then two post-match tests, but the authors do not effectively communicate the durations and specific time periods of data collection as these seem to be pertinent to their study.

**Response: We thank the reviewer for this comment. We revised the description of the study design and clarified all assessment time points in the Methods section and in Figure 1 to ensure consistency and transparency. The revised manuscript now clearly distinguishes pre-match, immediately post-match, and 24-, 48-, and 72-hour post-match assessments.**

Comment (Results):
The authors do not refer to the data in the figures while reporting the statistics of each variable.

**Response: We thank the reviewer for this observation. In the revised Results section, we improved the integration between the statistical reporting and the corresponding figures, so that the reader can more easily follow the results.**

Comment (Results):
There is inconsistent reporting of the periods of testing, and it becomes very difficult to determine if these data align with their purpose.

**Response: We thank the reviewer for this comment. We carefully revised the terminology and time-point labels throughout the manuscript to ensure consistency across the Abstract, Methods, Results, Discussion, Figures, and Table.**

Comment (Discussion):
The limitations of this study would seem to outweigh the ramifications of the results.

**Response: We appreciate this important comment. In the revised manuscript, we substantially softened the interpretation of the findings and the practical applications. We now present the results as preliminary observations from a specific ecological setting, and we explicitly acknowledge that the methodological limitations restrict the strength of the conclusions.**

Reviewer #3

Comment:
The study concerns “neuromuscular and subjective recovery in female volleyball players after match play”. The topic is relevant, the work is original, and the methods are generally sound. However, several major issues limit the strength of the findings and prevent the manuscript from meeting the publication standards required by PLOS ONE.

**Response: We sincerely thank the reviewer for recognizing the relevance and originality of the topic, as well as the general soundness of the methods. We also appreciate the clear identification of the major limitations. These comments were extremely helpful and led us to substantially revise the manuscript, especially regarding methodological transparency, the interpretation of the findings, and the practical conclusions.**

Major concerns

Comment:
The study includes only 12 players. They are then divided into two smaller subgroups (high play time; low play time). With such low numbers, especially when subjective measures are involved, the results cannot be generalised with confidence. Individual variation will strongly influence the findings. The sample is simply too small to support the conclusions drawn.

**Response: We fully agree with the reviewer that the sample size is a major limitation, particularly for subgroup analyses. In the revised manuscript, we have explicitly acknowledged this issue in the Methods, Discussion, and Conclusion sections, and we have substantially moderated the interpretation of the findings. We now present the results as exploratory and context-specific, and we avoid broad generalizations.**

Comment:
Moreover, there are concerns about reproducibility – recovery is influenced by many factors like sleep, nutrition, daily routines, stress, training load, match dynamics, etc. These were not controlled or fully reported. Without this information, the study is difficult to reproduce, and the results highly depend on context.

**Response: We thank the reviewer for this important observation. We agree that recovery is multifactorial and strongly influenced by contextual variables that were not fully controlled in the present study. In the revised manuscript, we acknowledged this limitation more explicitly and emphasized that the findings must be interpreted within the specific ecological context in which the data were collected. We also added discussion regarding the likely influence of contextual variables such as training load and match-related demands.**

Comment:
On page 13, two key CMJ strategy variables (contraction time & time to peak power) show poor ICC reliability (ICC < 0.5). Because of this, they cannot be used confidently to support claims about strategy or neuromuscular changes. Their low reliability undermines part of the interpretation.

**Response: We fully agree with the reviewer. In the revised manuscript, we substantially reduced the interpretative emphasis placed on these variables. We now explicitly state that contraction time and time to peak power showed poor reliability and, therefore, should be interpreted with caution. We also revised the Discussion and Conclusion to avoid strong claims based on these variables.**

Comment:
In my opinion, also use of median split is another issue. Dividing the sample into “high” and “low” play-time groups using median split is problematic especially with only 12 study participants. It reduces statistical power and not reflects meaningful physiological groups. This approach in my opinion strongly weakens the analysis.

**Response: Thank you for this comment. We acknowledge that the use of a median split in small samples can reduce statistical power and may not accurately reflect true physiological differences between groups. However, in this study the grouping based on playing time was not intended to establish distinct physiological categories, but rather to reflect a practical decision-making framework commonly used by coaching and performance staff in applied settings. In team sports such as volleyball, practitioners frequently dichotomize players (e.g., higher vs. lower match exposure) to guide load redistribution during the microcycle, particularly in MD+1 sessions. Therefore, the median split was used here as an operational proxy to reproduce this real-world approach within the analytical framework. To address this concern, we have revised the manuscript to clarify that this grouping strategy is context-driven and exploratory in nature, rather than a definitive classification of physiological status. We also explicitly acknowledge its limitations, including the potential loss of statistical sensitivity, and encourage future studies with larger samples to model playing time as a continuous variable or to apply more robust analytical approaches (e.g., mixed models) to better capture dose–response relationships.**

Comment:
In volleyball practice, the paper also recommends planning high-intensity sessions the day after a match. Given the small sample, low reliability of several variables, and context-dependent measures, these conclusions should be more cautious.

**Response: We fully agree. In the revised manuscript, we moderated the practical recommendations considerably. Rather than recommending high-intensity training on MD+1 in general terms, we now state that training prescription should be individualized and interpreted in light of recovery status, training context, and the cumulative effect of both match and subsequent training load.**

Minor comments

Comment:
Please define all abbreviations on first use. Terms such as RPE, MDC, CI, SEM, CV, RSI and ICC should be explained clearly when they first appear.

**Response: We thank the reviewer for this comment. All abbreviations were revised and are now defined at first use throughout the manuscript.**

Comment:
Similarly, thoroughly define all measured variables. All CMJ-related variables should be described fully to ensure reproducibility. Similar, TQR, HI and RPE depend heavily on how athletes are instructed. A brief explanation of the given instructions would strengthen the methods section.

**Response: We thank the reviewer for this valuable suggestion. The Methods section was revised to more clearly define the selected CMJ variables and to provide additional detail regarding the administration of TQR, HI, and RPE. This was done to improve reproducibility and methodological transparency.**

Reviewer #4

Comment:
The authors report on acute CMJ changes immediately and at 24 and 48 hrs following a volleyball match in a small group of semi-professional female players. The authors do not have the sample size necessary to be sub-grouping the cohort into low and high playing minutes, and in fact they need to be more cautious in their overall interpretation due to even the overall sample size being low for the types of interpretations and analysis performed.

**Response: We thank the reviewer for this important observation. We fully agree that the sample size limits both the subgroup analyses and the overall strength of the interpretations. In the revised manuscript, we have explicitly acknowledged this limitation, reduced the strength of our conclusions, and presented the findings as exploratory and context-specific.**

Specific comments

Comment (Abstract – Methods):
This study is not a longitudinal study as described in the methods; it is purely an acute single data series collection. Please remove the reference to a longitudinal study design.

**Response: We thank the reviewer for this clarification. The wording was revised accordingly, and references to a longitudinal design were removed. The study is now described more accurately as an acute repeated-measures design.**

Comment (Abstract – Conclusion):
This conclusion does not give enough consideration to the small sample size of this study.

**Response: We thank the reviewer for this comment. The conclusion in the Abstract was revised to reflect the exploratory nature of the findings and the limitations imposed by the sample size.**

Comment (Methods – Design):
As stated, the study is not longitudinal, but I agree that yes it is repeated measures of an acute response.

**Response: We thank the reviewer for this observation. The Methods section was revised accordingly to ensure that the study design is described accurately.**

Comment (Methods – Participants):
A sample size power calculation needs to be provided here to justify the inclusion of only an n=12 overall and that the sub-grouping analysis was sufficient to be performed with a 2-way ANOVA.

**Response: We thank the reviewer for this important comment. No a priori power analysis was conducted prior to data collection. We acknowledge this as a limitation of the study and have now explicitly stated this in the revised manuscript. In addition, we have substantially moderated the interpretation of the subgroup analyses and inferential results accordingly**

Comment (Methods – CMJ):
Can the authors please include the ICC, CV and smallest worthwhile change scores for these variables in this cohort of athletes so the reader can understand and interpret the magnitude of change relative to this cohort of athletes.

**Response: We thank the reviewer for this helpful suggestion. We reported ICC and CV values for the variables analyzed in this cohort. However, smallest worthwhile change was not included due to the limitations of the present sample size and study design. This has been acknowledged, and the interpretation of the magnitude of change has been made more cautious.**

Comment (Methods – Statistical Analysis):
As stated justify the sample size relative to this inferential statistical method or apply a different statistical approach that is less reliant on the sample size.

**Response: We appreciate this important methodological point. In the revised manuscript, we have more explicitly acknowledged the limitations of the inferential approach given the sample size and have tempered the interpretation of the analyses accordingly.**

Comment (Results):
There is data reported here of significant changes in the High minutes group at 24 and 48 hrs which does not align with statements made of no change in the abstract. The abstract needs to be adjusted to reflect that no change occurred only immediately post the match.

**Response: We thank the reviewer for this important observation. The Abstract was revised to better reflect the actual temporal pattern of the results. It now distinguishes between the immediate post-match time point and the subsequent post-match assessments.**

Comment (Discussion – 2nd Paragraph):
The opening sentence needs a time statement included as the match did not immediately post influence CMJ variables but there were changes observed at 24 and 48 hrs.

**Response: We thank the reviewer for this comment. The wording was revised accordingly to ensure that the temporal specificity of the findings is clearly stated.**

Comment (Conclusion):
Needs to be reworded to be more specific to the immediate post time point.

**Response: We thank the reviewer for this suggestion. The Conclusion was revised to be more temporally precise and to avoid overgeneralization across all post-match time points.**

Reviewer #5

Comment:
The present study explores how measures of jumping ability and well-being change due to match play and subsequent recovery in volleyball players. The paper is written concisely. There are, however, major concerns.

**Response: We sincerely thank the reviewer for the positive assessment of the manuscript’s conciseness and for the detailed and constructive comments. These observations helped us improve the manuscript substantially, particularly with respect to referencing, interpretation of the results, and the discussion of subjective measures and practical implications.**

Major concerns

Comment:
Many general statements regarding the use and effectiveness of athlete monitoring are made in the abstract and introduction without any proper referencing.

**Response: We thank the reviewer for this important observation. The Introduction was revised to ensure that general statements regarding athlete monitoring, performance, fatigue, and injury-related considerations are appropriately supported by the literature.**

Comment:
Results: A main effect of time is shown. However, this does not imply that values are different at all time points. Please give further analysis of which time points are in fact different.

**Response: We thank the reviewer for this comment. The Results section was revised to state more clearly which specific pairwise comparisons showed significant differences, rather than relying only on the main effect of time.**

Comment:
The discussion lacks mention of the subjective measures that were not different between time points and groups. Why was this not different?

**Response: We thank the reviewer for this valuable observation. The Discussion was expanded to address the absence of significant changes in subjective measures and to consider possible explanations, including the multidimensional nature of recovery, the limited sensitivity of these measures in the present context, and the possibility that subjective and neuromuscular responses may not follow identical temporal patterns.**

Comment:
The discussion seems to indicate different ideas about what a good time would be to do the next training session. This needs to be clearly explained.

**Response: We appreciate this important comment. We revised the Discussion to make the practical interpretation more coherent and cautious. The revised text now emphasizes that post-match training decisions should not be based solely on a single variable or time point, and that the apparent contradiction reflects the interaction between match load, subsequent training load, and recovery status. We now avoid a generalized recommendation and instead emphasize context-specific decision-making.**

Other minor concerns

Comment (Abstract):
‘Monitoring it helps optimize performance and prevent injuries’. I do not think this statement is necessarily valid in general and it should therefore not be part of the abstract.

**Response: We thank the reviewer for this comment. This statement was removed to avoid overgeneralization.**

Comment (Introduction):
Remove ‘…’ after serve.

**Response: We thank the reviewer for this observation. This was corrected.**

Comment (Introduction):
‘To maximize performance, it is necessary to monitor athletes with the highest possible frequency...’ This is not true.

**Response: We thank the reviewer for this important point. The sentence was revised to avoid an overgeneralized statement and to better reflect the complexity of athlete monitoring practice.**

Comment (Introduction):
‘The benefits of monitoring athletes are numerous...’ Provide references.

**Response: We thank the reviewer for this observation. Appropriate references were added and the wording was revised for greater precision.**

Comment (Introduction):
‘Jumping ability is a sensitive marker of fatigue...’ Again, this is a statement without evidence.

**Response: We thank the reviewer for this comment. The statement was revised and supported with appropriate references.**

Comment (Introduction):
‘The analysis of alternative metrics to traditional ones is an excellent method...’ What are the alternative vs traditional metrics?

**Response: We thank the reviewer for this suggestion. This section was revised to clarify what was meant by traditional versus alternative CMJ metrics.**

Comment (Introduction):
Last line: TQR and HI were initially mentioned, and then only TQR. Is there a reason for this selection?

**Response: We thank the reviewer for identifying this inconsistency. The text was revised to ensure consistent reference to both subjective measures and to clarify their inclusion in the study.**

Comment (Methods – Design):
No mention of TQR and when it was measured. Please add.

**Response: We thank the reviewer for this comment. The timing of TQR and HI administration was clarified in the revised Methods section.**

Comment (Methods – Design):
Can the authors briefly explain why PCA is specifically used?

Comment (Warm-up):
‘The intensity of the warm-up progressively increased’ It is unclear what this means.

**Response: We thank the reviewer for this observation. The description of the warm-up was revised to clarify that the intensity increased progressively within each warm-up sequence, not across different post-match testing days.**

Comment (Training and competition loads quantification):
Change ‘will be used’ to ‘was used’.

**Response: We thank the reviewer for identifying this tense inconsistency. This was corrected.**

Comment (Statistical Analysis):
Nowhere in the introduction or aim of the study was playing time mentioned.

**Response: We thank the reviewer for this important observation. The aim of the study was revised to better explain the consideration of playing time in the analysis.**

Comment (Results):
A main effect of time does not show which times were different.

**Response: We thank the reviewer for this comment. The Results section was revised to specify the pairwise comparisons that reached statistical significance.**

Comment (Discussion):
The discussion has very little mention of the fact that TQR and HI results did not change at all. Please address this more.

**Response: We thank the reviewer for this valuable comment. We expanded the Discussion to more fully address the lack of change in TQR and HI and to place these results in the context of previous literature and the multidimensional nature of recovery.**

Comment (Discussion):
There appears to be a contradiction between recommending 24–48 h after competition and recommending MD+1 training.

**Response: We thank the reviewer for this important comment. We agree that the previous wording could be interpreted as contradictory between recommending 24–48 h after competition and MD+1 training. To avoid any inconsistency, we have revised the manuscript to unify the practical recommendation. Specifically, we now consistently refer to MD+1 as the main reference point for post-competition training resumption, which corresponds to the early recovery window (approximately 24 h after competition). This clarification improves consistency and better aligns the recommendation with the observed recovery timeline.**
