# Supplementary material for: Monitoring CMJ-derived metrics and subjective recovery across a competitive microcycle in semi-professional women’s volleyball
Source: PLoS One. 2026 Sep 1;21(9):e0328664. doi: 10.1371/journal.pone.0328664 (PMC13533353; doi:10.1371/journal.pone.0328664)
Supplement: Supplementary file 4 [file pone.0328664.s003.docx]

**Letter of response**

**Manuscript: [PONE-D-25-34659R1] - [EMID:a033410873d87333]**

Reviewer #4: The authors have made substantial changes to the manuscript in response to the first round review which has significantly improved the integrity and reproducibility. The are however some grammatical errors and the interpretation can be clarified with some adjustments. In particular the authors should rewrite any sentences using an en dash as a writing emphasis, as it is poor quality lazy writing.

Specific Comments
Ln 60-63; Reword to remove the use of the en dash

***Authors: We thank the reviewer for this comment. The use of en dashes has been revised throughout the manuscript, and they have been replaced accordingly to ensure consistency with the journal’s style requirements.***
Ln 65; This is not the start of new paragraph and the word ‘assists’ should not be plural.

***Authors:*** ***We thank the reviewer for this comment. The text has been revised accordingly: the paragraph structure has been corrected, and the verb has been adjusted by removing the plural form to ensure grammatical accuracy.***
Ln 66; change ‘its’ to ‘of’

***Authors:*** ***We thank the reviewer for this suggestion. The wording has been corrected as recommended, replacing “its” with “of” to improve grammatical accuracy and clarity.***
Ln 69; Delete ‘This assessment of’ and replace with, ‘Assessing…’

***Authors:*** ***We thank the reviewer for this suggestion. The sentence has been revised as recommended by deleting “This assessment of” and replacing it with “Assessing…” to improve clarity and conciseness.***
Ln 74; Insert ‘CMJ’ so the sentence reads, “….alternative CMJ metrics represents an effective approach…”

***Authors: We thank the reviewer for this suggestion. The sentence has been revised accordingly by inserting “CMJ” so that it now reads: “…alternative CMJ metrics represents an effective approach…”.***
Ln 79; Delete the word ‘their’

***Authors: We thank the reviewer for this suggestion. The word “their” has been deleted as recommended to improve grammatical clarity.***
Ln 91; Poor grammar re word, delete the words “It has” and replace with, “For example the HI has shown…”

***Authors: We thank the reviewer for this indication. The sentence has been revised accordingly by deleting “It has” and replacing it with “For example, the HI has shown…” to improve grammatical accuracy and clarity.***
Ln 93; poor grammar, re-word and combine these two sentences so that it reads,… “72 hours post-match, supporting the use and combination of subjective assessments….”

***Authors:*** ***We thank the reviewer for this helpful comment. The sentence has been reworded and combined as recommended to improve grammatical flow and clarity, now reading: “…72 hours post-match, supporting the use and combination of subjective assessments…”.***
Ln 97; Add space after ‘sports’

***Authors: We thank the reviewer for this observation. The space has been added.***

Ln 239; I agree with the author’s statement but I suggest adding a further clarifying comment, to the effect of, "However match plus training loads on MD+1 does significantly impact on jump metrics at subsequent timepoints pointing towards an accumulative effect of high neuromuscular loads."

***We thank the reviewer for this valuable suggestion. We agree that this additional clarification strengthens the interpretation of our findings. Accordingly, we have incorporated the following statement into the Discussion:***

***"However, the combination of match and training loads on MD+1 appears to influence subsequent jump performance, suggesting a cumulative effect of high neuromuscular loading across the microcycle."***

***We appreciate this comment, which has improved the interpretation of our findings.***

***Authors:***
Ln 239; While understand why the authors have used the term "In contrast" here, if the suggested added statement above is included I do not believe that this is necessary and the idea flows better to the comparison to other sports.

***We appreciate this suggestion. Following the inclusion of the additional explanatory statement above, we have removed the expression "In contrast" to improve the logical flow and readability of the paragraph.***

***Authors:***
Ln 255-263; I agree with this added interpretation however I suggest the authors consider the work of Hughes et al (2021) JSCR as one available example to support this discussion point.

***We thank the reviewer for this excellent suggestion. The study by Hughes et al. (2021) has now been incorporated into the Discussion to further support our interpretation regarding the cumulative neuromuscular effects observed following competition and subsequent training exposure. We believe that the inclusion of this reference strengthens the scientific context of our findings.***

***Authors:***
Ln 269-272; I agree with this point but as suggested this factor could be introduced or at the least alluded to earlier.

***Authors: We appreciate this observation. We agree that introducing this concept earlier improves the coherence and progression of the Discussion. Accordingly, we have incorporated a statement in the preceding paragraph acknowledging that the cumulative neuromuscular stimulus differed between subgroups due to their different exposure to match play and subsequent training. We believe this addition provides a clearer framework for interpreting the observed CMJ responses throughout the microcycle.***

Ln 274-275; Poor grammar and use of the en dash, reword, "...time, who in contrast were those exposed...MD+1, ..."

***Authors: We thank the reviewer for this helpful correction. The sentence has been reworded to improve grammatical accuracy and clarity, and the en dash has been removed. It now reads: “…time, who in contrast were those exposed to MD+1…”.***
Ln 276; Suggest a wording change, delete the words ‘again showed’ and replace as, “…impulse, recorded maximal…”

***Authors: We thank the reviewer for this helpful suggestion. The sentence has been revised as recommended by deleting “again showed” and replacing it with “recorded maximal…”, improving both clarity and conciseness.***
Ln 281-282; Poor grammar and use of the en dash, reword, "..metrics, such as concentric impulse is enhanced, reinforcing the value of this metric for..."

***Authors: We thank the reviewer for this helpful suggestion. The sentence has been reworded to improve grammatical structure and clarity, and the en dash has been removed. It now reads: “…metrics, such as concentric impulse is enhanced, reinforcing the value of this metric for…”.***
Ln 290; Change the word ‘showed’ to ‘recorded’. It is not possible for a variable to have an action. Delete the word ‘which’, include a comma after the citation and change ‘suggests’ to ‘suggesting’

***Authors: We thank the reviewer for this helpful observation. The sentence has been revised as recommended: “showed” has been replaced with “recorded,” “which” has been deleted, a comma has been added after the citation, and “suggests” has been changed to “suggesting” to improve grammatical accuracy and flow.***
Ln 317; Change “This stability…’ to “The stability of response in these measures suggests…”

***Authors:*** ***We thank the reviewer for this helpful suggestion. The sentence has been revised as recommended by changing “This stability…” to “The stability of response in these measures suggests…” to improve clarity and grammatical flow.***
Ln 318; Change ‘by’ to ‘in’

***Authors: We thank the reviewer for this helpful correction. The preposition has been amended as suggested, replacing “by” with “in” to improve grammatical accuracy.***
Ln 319; Add (HI) after measures

***Authors: We thank the reviewer for this helpful suggestion. The abbreviation (HI) has been added after “measures” as recommended to improve clarity and ensure consistency in terminology.***
Ln 326; Poor grammar and use of the en dash, reword.

***Authors: We thank the reviewer for this helpful correction. The sentence has been reworded to improve grammatical accuracy, and the en dashes have been removed and replaced with commas as appropriate to ensure consistency and clarity throughout the manuscript.***
Ln 348; Remove the word 'may' and make the word ‘limit’ plural.

***Authors: We thank the reviewer for this helpful suggestion. The sentence has been revised by removing “may” and changing “limit” to the plural form “limits” to improve grammatical accuracy and clarity***
Ln 348-349; Add a statement after this sentence, "However it is not ecologically or ethically valid in this applied setting to include a control condition."

***Authors: We thank the reviewer for this insightful suggestion. The sentence has been updated accordingly, and a clarifying statement has been added as recommended: “However, it is not ecologically or ethically valid in this applied setting to include a control condition.” We appreciate this valuable comment, which has strengthened the interpretation of the study limitations.***
Ln 351; Add after the word ‘addition’, ‘to this point’

***Authors: We thank the reviewer for this helpful suggestion. The text has been revised by adding “to this point” after “addition” to improve clarity and flow.***
Ln 352; Insert after the word ‘influenced’ and delete the word ‘both’ “..our ability to fully interpret the…”

***Authors: We thank the reviewer for this helpful suggestion. The sentence has been revised accordingly by inserting “our ability to fully interpret the…” after “influenced” and deleting “both” to improve grammatical accuracy and clarity.***
Ln 355; Insert the following after the word “…considered,” “due to our limited sample size, however this factor in future investigations may highlight potential differences...

***Authors: We thank the reviewer for this helpful suggestion. The sentence has been revised as recommended by inserting the following text after “considered,”: “due to our limited sample size, however this factor in future investigations may highlight potential differences…”, in order to improve clarity and strengthen the discussion of limitations and future directions.***

Reviewer #6: This study aims to analyse biomechanical CMJ variables in semi-professional female volleyball players before, immediately after and 24, 48, 72 hours following a competitive match play. Although the authors acknowledge several limitations, there is one fundamental issue that cannot be adequately resolved through limitation statements alone. the title and abstract lead the reader to expect an analysis of CMJ variations attributable to match play and subsequent recovery. However, this interpretation is problematic because the athletes performed a training session at MD+1. Consequently, comparisons between pre-match values and measurements obtained after this training session cannot be interpreted as reflecting the isolated effects of match play or recovery from competition alone. Moreover, the manuscript does not report whether athletes instructions regarding recovery-related activities, such as stretching, additional physical activity or other at-home practices. Together with the inclusion of the training session and all other limitations listed in the manuscript, these uncontrolled factors make it difficult to attribute CMJ variations specifically to match-induced fatigue and recovery.

***Authors: We thank the reviewer for this thoughtful and constructive comment. We agree that the inclusion of the MD+1 training session, together with the inability to control individual recovery-related behaviours, prevents attributing the observed CMJ responses exclusively to match play or recovery from competition.***

***Our intention was not to isolate the physiological effects of match play, but rather to describe the neuromuscular responses occurring throughout a real competitive microcycle under ecological conditions. To better reflect this perspective, we have revised the title, abstract, objectives, discussion, and conclusions to avoid implying causal interpretations related solely to match-induced fatigue or recovery. In addition, we have expanded the limitations section to explicitly acknowledge the potential influence of both the scheduled training session and uncontrolled recovery behaviours on the observed responses.***

***Now it reads: An important consideration is that the observed changes cannot be attributed exclusively to match play, as players also completed a scheduled training session on MD+1 and individual recovery-related behaviours outside the team environment (e.g., stretching, additional physical activity, or other recovery strategies) were not controlled. Consequently, the present findings should be interpreted as the neuromuscular responses occurring throughout a real competitive microcycle rather than as isolated effects of competition or recovery”***

***We sincerely thank the reviewer for this valuable comment, which has improved the transparency and interpretation of our study.***

Reviewer #7: The study attempts to quantify neuromuscular fatigue and performance in experienced women’s volleyball players via the counter movement jump (CMJ), rate of perceived exertion (RPE), total quality recovery scale (TQR), and the Hooper Index (HI). Whereas variables associated with CMJ are related to objective measures such as jump height, contraction time, or time to peak power, the other measures are somewhat subjective.

The study is timely in that a number of other researchers have shown an interest in using metrics associated with the CMJ as gauges of neuromuscular fatigue, and the author cites a number of them throughout the paper. There is especially acute interest in volleyball and in other jumping sports as CMJ related metrics can help to decouple performance measures like jump height from how an athlete moves to obtain that result. The authors, in my opinion, describe the motivation for the study quite clearly. However, the science presented is incremental, and the authors need to explain clearly what they are bringing to the table (validation of other results in volleyball, indication that such results apply to a more elite women’s group, usage of TQR+HI in conjunction with CMJ measures?). Likewise, I concur with previous reviewers regarding the grouping – while it seems to be an attempt to control for loading, it may result in under-powered statistics. Potentially using a single group and focussing on the time response could make those results clearer.

This is at least their second attempt at publication, and it’s clear that they have tried to address previous reviewers’ comments in this draft. What follows are some specific comments, some more critical than others. I’ve put an asterisk* by the important ones.

*1) Abstract, Lines 32-33: “Significant changes were observed … in players with less playing time.” Is this true? The figures at the end of the paper don’t seem to indicate that this is the case. Later RSIMod and Concentric Impulse are noted to have significant differences (lines 217-219), but Figure 3 shows no notational indication of this.
***Authors: We thank the reviewer for this important observation. We have updated the corresponding figure to ensure consistency with the statistical results presented in the manuscript. The figures now accurately reflect the significant differences reported in the abstract.***

2) Line 64 – spacing. “fatigue experienced by the athlete. [space needed] In this context, the CMJ…”

***Authors: Thanks to the reviewer for this observation. We have added the needed space.***

3) Line 121 – If you are abbreviating match day as MD, be consistent. You have here: “...the morning of each match day, MD+1, Md+2 and MD+3.” Your second MD is mixed case.
***Authors: We thank the reviewer for identifying this inconsistency. The abbreviation has now been corrected throughout the manuscript, and all references to match day are consistently reported in uppercase format (MD, MD+1, MD+2, and MD+3). We appreciate this observation, which has improved the consistency of the manuscript.***
4) Line 142 – General style standards hew to APA (but the editors may feel this is not important). Numbers less than 10 should be spelled out, unless there’s a good reason not to (for example when specifying a range). “Participants performed three attempts…”
***Authors: We thank the reviewer for this helpful observation. In accordance with APA style recommendations, numbers lower than ten have been revised from numerals to words where appropriate. Specifically, three (line 138), one (line 140), and ten (line 145) have been written in full to improve consistency with the journal's style guidelines.***
5) Lines 150-154 – I’m not a big fan of the list presentation. I’d prefer to see these in paragraph form OR in a table, not as an unmarked list. I’ll defer to the editors again on this.
***Authors:*** ***We thank the reviewer for this suggestion. Following the recommendation, the information previously presented as a list has been reformatted into paragraph form to improve readability and maintain consistency with the style of the manuscript. The revised text now describes the analysed variables within a continuous narrative format. We appreciate this comment, which has helped improve the presentation of the methods section (lines 146-151).***
6) Line 174 – “scores of the 4 questionnaire items” → “scores of the four questionnaire items” - again this is editorial preference.
***Authors: We thank the reviewer for this observation. In accordance with the editorial style recommendations, the numeral has been replaced with the corresponding word, and the text now reads “scores of the four questionnaire items.”***
*7) Line 180 – Weir’s 2005 paper makes a point of breaking down several different ICCs in the Shrout and Fleiss framework and which is appropriate to a given study. The question is: are you looking for real consistency (ICC(3,1)) or are you looking for absolute agreement (ICC(2,1))? You should specify more clearly what you’re after from your ICCs because they will give you very different values dependent on which one you use.
***Authors: We thank the reviewer for this important observation. We agree that the specific ICC model should be clearly stated, as different forms of ICC address different aspects of reliability. Accordingly, we have revised the manuscript to specify that a two-way mixed-effects model assessing consistency (ICC(3,1)) was used, following the recommendations of Shrout and Fleiss and Weir (2005).***
8) Line 186 – “CI95%” should probably be “CI 95%”.
***Authors:*** ***We thank the reviewer for this comment. The notation has been corrected, and “CI95%” has been revised to “CI 95%” throughout the manuscript to ensure consistency with standard reporting conventions.***
*9) Line 191 – “The Bonferroni correction was applied for post-hoc correction”. The Bonferroni correction is known to be overly conservative in most cases. While it reduces the chance of false positives, it also increases the chance of false negatives – often more than is warranted. Unless you have a good reason for using it (for example you didn’t do every possible post-hoc), I would go with Holm-Bonferroni or possibly Tukey to avoid overly increasing the possibility of false negatives. In short, please give a good explanation for your choice of this stat since it could affect your findings.
***Authors: We thank the reviewer for this valuable comment. We agree that Holm-Bonferroni and Tukey procedures may provide greater statistical power under certain circumstances. However, the Bonferroni correction was selected a priori because the number of planned pairwise comparisons was limited and predefined according to our repeated-measures design.***

***Given the relatively small sample size and the exploratory nature of this study, we considered a conservative approach appropriate to minimise the probability of Type I error.***

*10) Line 197 – The jump height ICC is suspicious in that its interval values range from 0.01-0.99 rendering the value of ICC = 0.98 nearly meaningless. In fact, the confidence interval may be more meaningful than the ICC in this case. Note that with small sample sizes, the ICC can be really unstable in that a small change in one athlete’s score could substantively change the ICC value. This suggests that the authors should be very careful interpreting this result and should not say on line 199 that it “demonstrated excellent reliability”.
***Authors: We thank the reviewer for this important comment and for highlighting the need for a more cautious interpretation of the ICC results. Following this suggestion, we have revised the manuscript and replaced the original wording with “showed good to excellent reliability, although jump height with wide confidence intervals,” to better reflect the uncertainty associated with the estimate.***
11) Lines 207-219 – If ES is intended to be the Cohen d main effect size, you should indicate that this is what it is before using this abbreviation. Apologies if I missed the definition.
***Authors: We thank the reviewer for this helpful comment. We have now added the corresponding definition in the Methods section, clarifying that ES refers to Cohen’s d as the main effect size used in the analyses.***
*12) Line 218 – You publish a concentric impulse effect size of (ES = -0.60 [-2.67 to -1.48]). Your central value is not in the interval. This needs explanation as it’s statistically implausible.

***Authors: We thank the reviewer for carefully identifying this inconsistency. We have reviewed the reported values and confirmed that the effect size estimate was correct (ES = -0.60), but the associated confidence interval was incorrectly reported due to a transcription error. The confidence interval has now been corrected in the revised manuscript to ensure that it accurately corresponds to the reported effect size. We appreciate the reviewer’s careful reading, which helped us identify and rectify this mistake.***

***Authors:***
*13) Lines 239-242 – “All these findings suggest … allowing for high-load on the following sessions (MD+1)”. I disagree. Delayed onset muscle soreness (DOMS) is a real result of eccentric loading which could plausibly explain the decrease in performance of athletes after increased loading. The onset of DOMS can range from 6-24 hours and can peak between 1-3 days after the activity that caused it. I strongly suspect that a hard training day post-game will lead to longer down time for athletes later. I feel that your statement confuses the ability to train (“I don’t feel sore yet”) with best training practices. See these sources below. They may play a role in your discussion.

***Authors: We thank the reviewer for this thoughtful and constructive comment. We agree that the absence of immediate neuromuscular impairment should not be interpreted as evidence supporting the implementation of high-load training on MD+1. As the reviewer correctly points out, delayed fatigue responses, including delayed onset muscle soreness (DOMS) and other manifestations of accumulated neuromuscular stress, may develop during the 24–72 hours following competition and should be considered when interpreting the temporal responses observed across the microcycle.***

***Accordingly, we have revised the Discussion to avoid this potential overinterpretation. The manuscript now emphasises that our findings describe the neuromuscular responses observed under the specific conditions of a real competitive microcycle, where both match exposure and the subsequent MD+1 training session contribute to the cumulative loading experienced by the players, rather than supporting specific recommendations regarding training load prescription. Furthermore, we have incorporated the suggested literature to strengthen the discussion of the potential delayed effects of accumulated neuromuscular loading.***
