# Supplementary material for: Monitoring CMJ-derived metrics and subjective recovery across a competitive microcycle in semi-professional women’s volleyball
Source: PLoS One. 2026 Sep 1;21(9):e0328664. doi: 10.1371/journal.pone.0328664 (PMC13533353; doi:10.1371/journal.pone.0328664)
Supplement: Supplementary file 5 [file pone.0328664.s005.docx]

**Response to Reviewers**

*Monitoring CMJ-Derived Metrics and Subjective Recovery Across a Competitive Microcycle in Semi-Professional Women's Volleyball*

Dear Editor and Reviewers,

We thank the Editor and the Reviewers for their careful assessment of the revised manuscript. We have addressed all remaining comments and conducted a final consistency and formatting check. The revisions are intentionally focused and do not alter the study design, analyses, principal findings, or overall conclusions. Our point-by-point responses are provided below. Reviewer comments are reproduced in bold, followed by our responses and the corresponding changes made in the manuscript.

# Reviewer #4

**The authors have made substantial changes to the manuscript following the first review round; however, there are discrepancies between the reported changes and the actual manuscript changes. In particular, the previously stated inclusion of Hughes et al. (2021) was not present. The remaining discussion of delayed or prolonged neuromuscular fatigue should also be supported by primary evidence rather than relying mainly on the general review by Bishop et al. (2023). The authors should ensure consistency between the response letter and the revised manuscript.**

**Response:** We agree with the Reviewer and apologize for the discrepancy between our previous response and the revised manuscript. During the substantial restructuring of the Discussion, the sentence containing the citation to Hughes et al. was inadvertently removed. We have now corrected this inconsistency. The study by Hughes et al. has been incorporated into the Discussion as primary experimental evidence showing that fatigue-related changes can be detected across the CMJ force-time curve and may not be fully represented by jump height alone. In addition, we revised several statements concerning delayed neuromuscular fatigue so that they are framed more cautiously and linked directly to the evidence available from the present data and primary studies.

# Reviewer #8

**1. Consistency in Decimal Places (L122, Table 1, etc.). Inconsistencies in the number of decimal places were observed. Please standardize these throughout the manuscript, taking into account the appropriate number of significant figures for each metric.**

**Response:** We agree. The numerical values in Table 1 have been standardized and rounded to zero decimal places to ensure consistent reporting within the table. inferential statistics and reliability coefficients to two decimal places where appropriate, and p values to three decimal places, except when p < 0.001.

**2. Clarification of Jump Height ICC Confidence Interval (L194). Is the reported 95% CI for the jump height ICC (0.01–0.99) correct? Given that the ICC is 0.98, this interval appears unusually wide. Please double-check this value.**

**Response:** We thank the Reviewer for identifying this issue. The lower confidence limit was a transcription error. We rechecked the reliability output and corrected the confidence interval in the manuscript.

**3. Reporting Effect Sizes for ANOVA (L204–205). For the ANOVA results, include an effect size such as partial eta squared (ηp²).**

**Response:** We agree and have added partial eta squared values for the significant main effects of time.

**4. Wording for Significant Differences (L209). Add the word “significant” before “reductions.”**

**Response:** We agree and have revised the sentence accordingly.

**5. Notation of Effect Sizes in the Results Section (L210, etc.). Since Cohen’s d was calculated, use “d = XX” rather than the generic abbreviation “ES.”**

**Response:** We agree. The generic abbreviation “ES” has been replaced by Cohen’s d notation throughout the Results and in the relevant parts of the Discussion.

**6. Clarification of “each” (L252). The phrase “the duration of each point” is ambiguous.**

**Response:** We agree and have clarified that the term refers to each rally.

**7. Consistent Use of Abbreviations (L263). Since minimal detectable change has already been defined, use “MDC” only.**

**Response:** We agree and have removed the repeated definition.

**8. Spacing and Formatting (L308, etc.). Conduct a thorough formatting review.**

**Response:** We agree. We conducted a complete proofread and corrected missing or extra spaces, punctuation, unit formatting, and typographical errors throughout the manuscript. PENDIETEEEE

**9. Enhancement of Figure Captions (L505–506). The captions for Figures 1 and 2 are too brief. Please provide more descriptive captions.**

**Response:** We agree. All figure captions have been expanded so that the figures can be interpreted independently from the main text. The revised captions define the time points, groups, symbols, summary statistics, error bars, and significance annotations.
