# Supplementary material for: Monitoring CMJ-derived metrics and subjective recovery across a competitive microcycle in semi-professional women’s volleyball
Source: PLoS One. 2026 Sep 1;21(9):e0328664. doi: 10.1371/journal.pone.0328664 (PMC13533353; doi:10.1371/journal.pone.0328664)
Supplement: Peer review history

# Peer review: Decision Letter 0

**DOI:** 10.1371/journal.pone.0328664.r001

**Authors:** Mário Espada

24 Nov 2025

Monitoring jumping ability and subjective recovery in women's volleyball: acute effects of match play on countermovement jump and well-being

PLOS ONE

Dear Dr. González-García,

Thank you for submitting your manuscript to PLOS ONE. After careful consideration, we feel that it has merit but does not fully meet PLOS ONE’s publication criteria as it currently stands. Therefore, we invite you to submit a revised version of the manuscript that addresses the points raised during the review process.

Please submit your revised manuscript by Jan 05 2026 11:59PM. If you will need more time than this to complete your revisions, please reply to this message or contact the journal office at plosone@plos.org. When you're ready to submit your revision, log on to https://www.editorialmanager.com/pone/ and select the 'Submissions Needing Revision' folder to locate your manuscript file.

- A rebuttal letter that responds to each point raised by the academic editor and reviewer(s). You should upload this letter as a separate file labeled 'Response to Reviewers'.
- A marked-up copy of your manuscript that highlights changes made to the original version. You should upload this as a separate file labeled 'Revised Manuscript with Track Changes'.
- An unmarked version of your revised paper without tracked changes. You should upload this as a separate file labeled 'Manuscript'.

If you would like to make changes to your financial disclosure, please include your updated statement in your cover letter. Guidelines for resubmitting your figure files are available below the reviewer comments at the end of this letter.

If applicable, we recommend that you deposit your laboratory protocols in protocols.io to enhance the reproducibility of your results. Protocols.io assigns your protocol its own identifier (DOI) so that it can be cited independently in the future. For instructions see: https://journals.plos.org/plosone/s/submission-guidelines#loc-laboratory-protocols. Additionally, PLOS ONE offers an option for publishing peer-reviewed Lab Protocol articles, which describe protocols hosted on protocols.io. Read more information on sharing protocols at https://plos.org/protocols?utm_medium=editorial-email&utm_source=authorletters&utm_campaign=protocols.

We look forward to receiving your revised manuscript.

Kind regards,

Mário Espada, PhD

Academic Editor

PLOS ONE

Journal Requirements:

When submitting your revision, we need you to address these additional requirements.

1. Please ensure that your manuscript meets PLOS ONE's style requirements, including those for file naming. The PLOS ONE style templates can be found at

https://journals.plos.org/plosone/s/file?id=wjVg/PLOSOne_formatting_sample_main_body.pdf and https://journals.plos.org/plosone/s/file?id=ba62/PLOSOne_formatting_sample_title_authors_affiliations.pdf

2. Please note that your Data Availability Statement is currently missing the repository name or a direct link to access each database. If your manuscript is accepted for publication, you will be asked to provide these details on a very short timeline. We therefore suggest that you provide this information now, though we will not hold up the peer review process if you are unable.

3. When completing the data availability statement of the submission form, you indicated that you will make your data available on acceptance. We strongly recommend all authors decide on a data sharing plan before acceptance, as the process can be lengthy and hold up publication timelines. Please note that, though access restrictions are acceptable now, your entire data will need to be made freely accessible if your manuscript is accepted for publication. This policy applies to all data except where public deposition would breach compliance with the protocol approved by your research ethics board. If you are unable to adhere to our open data policy, please kindly revise your statement to explain your reasoning and we will seek the editor's input on an exemption. Please be assured that, once you have provided your new statement, the assessment of your exemption will not hold up the peer review process.

4. Please upload a new copy of Figure 4 as the detail is not clear. Please follow the link for more information:  https://journals.plos.org/plosone/s/figures

5. Please include captions for your Supporting Information files at the end of your manuscript, and update any in-text citations to match accordingly. Please see our Supporting Information guidelines for more information: http://journals.plos.org/plosone/s/supporting-information.

If the reviewer comments include a recommendation to cite specific previously published works, please review and evaluate these publications to determine whether they are relevant and should be cited. There is no requirement to cite these works unless the editor has indicated otherwise.

Additional Editor Comments:

Dear Authors,

Congratulations on your work.

Please revise the manuscript in light of the reviewers' suggestions.

Thank you.

Best regards.

[Note: HTML markup is below. Please do not edit.]

Reviewers' comments:

Reviewer's Responses to Questions

**Comments to the Author**

1. Is the manuscript technically sound, and do the data support the conclusions?

Reviewer #1: Partly

Reviewer #2: Yes

Reviewer #3: Partly

Reviewer #4: Partly

Reviewer #5: Partly

**********

2. Has the statistical analysis been performed appropriately and rigorously?

Reviewer #1: No

Reviewer #2: Yes

Reviewer #3: Yes

Reviewer #4: No

Reviewer #5: No

**********

3. Have the authors made all data underlying the findings in their manuscript fully available?

The PLOS Data policy

Reviewer #1: Yes

Reviewer #2: Yes

Reviewer #3: Yes

Reviewer #4: Yes

Reviewer #5: Yes

**********

4. Is the manuscript presented in an intelligible fashion and written in standard English?

Reviewer #1: No

Reviewer #2: Yes

Reviewer #3: Yes

Reviewer #4: Yes

Reviewer #5: Yes

**********

Reviewer #1: Thank you for giving me the opportunity to review your manuscript.

This is an interesting study that is likely to interest some readers. However, in my opinion it requires extensive revision. There is a lot of repetition, and much of the phrasing is confusing which in turn compromises the clarity and accessibility of key points.

I also think that some results have been interpreted poorly, and the rationale for choosing some of the dependent variables is questionable.

For example, what is the rationale for considering time to peak power but not studying the mean net force and time elements of the concentric impulse?

Given the relatively small sample size and poor reliability, should the time to peak power and time to takeoff results be discussed so boldly? I would strongly argue that far more caution should be exercised by the authors and that readers should be urged to exercise caution when considering these variables.

With the reliability in mind, what data was used to calculate this because your figures, which are excellent, clearly show increases in variability which would influence reliability and potentially explain some of your key results.

I have provided more specific feedback in the attached, annotated PDF.

Reviewer #2: Summary:

Biomechanical variables involved in jump mechanics of semi-professional level female volleyball players were evaluated before, immediately after one day after, two days after, and three days after match play.

Comments:

The authors have a good premise for assessing female volleyball players. How does the neuromuscular system recovery after a match in regards to counter-movement jump performance? There are a number of limitations which the authors acknowledge for this study. The largest limitation is the grouping of players into high match-load versus lower match load. With an N = 12, this significantly reduces the statistical power. The authors also did not make it clear in the text of the document how many players were in each group. It is also not clear how the training cycle influenced these results. The authors discuss the cycles of training, but this was not mentioned as a mitigating factor in this study.

Another aspect of the study which is not mentioned is the positioning of the players. Typically, in a volleyball match the front line players jump more frequently than the other players on the court. This was not a factor addressed by this manuscript. The lack of a control group and the number of participants makes it difficult to readily evaluate the overall meaning of these data.

Introduction

This section seems to be very well documented and provides good literature to support the purpose of the study.

Methods

- How specific are the demographic information? It seems that if height was measured in cm, then a standard deviation of 6 cm will suffice and not 6.01 cm, likewise, the standard deviations for the age and mass should be to one decimal point

- If N = 12 and there was a comparison between groups – and possibly uneven numbers between groups, the statistical power would seem to be lowered. Was a power analysis conducted prior to the initiation of the study? The authors do not provide the number of participants for each group

- An explanation of the time periods of data collection are not clear. A pre-test was performed and then two post-match tests, but the authors do not effectively communicate the durations and specific time periods of data collection as these seem to be pertinent to their study

Results

- The authors do not refer to the data in the figures while reporting the statistics of each variable

- There is inconsistent reporting of the periods of testing, and it becomes very difficult to determine if these data align with their purpose

Discussion

- The limitations of this study would seem to outweigh the ramifications of the results

Reviewer #3: Comments to the autkors

The study concerns “neuromuscular and subjective recovery in female volleyball players after match play”. The topic is relevant - agrees with most of PLOS1 criteria for publication (given by Guidelines for Reviewers), and the work is original. The methods are generally sound, and the paper is clearly structured. However, several major issues limit the strength of the findings and prevent the manuscript from meeting the publication standards required by PLOS 1.

Major concerns

- The study includes only 12 players. They are then divided into two smaller subgroups (high play time; low play time). With such low numbers, especially when subjective measures are involved, the results cannot be generalised with confidence. Individual variation will strongly influence the findings. The sample is simply too small to support the conclusions drawn.

- Moreover, thera are concerns about reproducibility – the recovery is influenced by many factors likesleep, nutrition, daily routines, stress, training load, match dynamics,…. These as I read were not controlled or fully reported. Without this information, the study is difficult to reproduce, and the results highly depend on context.

- On page 13, two key CMJ strategy variables (contraction time & time to peak power) show poor ICC reliability (ICC < 0.5 – or even -0.24). Because of this, they cannot be used confidently to support claims about strategy or neuromuscular changes. Their low reliability undermines part of the interpretation.

-In my opinion, also use of median split is another issue. Dividing the sample into “high” and “low” play-time groups using median split is problematic especially with only 12 study participants. It reduces statistical power and not reflects meaningful physiological groups. This approach in my opinion strongly weakens the analysis.

- In volleyball practice, the paper also recommends planning high-intensity sessions the day after a match. Given the small sample, low reliability of several variables, and context-dependent measures, these conclusions should be more cautious. The work provides interesting observations, but it cannot support practical training recommendations in its current form.

For these reasons, my recommendation is REJECT. I encourage the authors to consider repeating the study with a larger cohort and a more controlled design. The topic is worthwhile, and a stronger dataset could lead to more meaningful and generalisable findings.

Minor comments to be useful in future, include:

Please, define all abbreviations on first use. Terms such as RPE, MDC, CI, SEM, CV, RSI and ICC should be explained clearly when they first appear. This helps clarity and accessibility for all readers.

Similarly, thoroughly define all measured variables. All CMJ-related variables should be described fully to ensure reproducibility. Some definitions are present, but others are missing or incomplete. Similar, TQR, HI and RPE depend heavily on how athletes are instructed. A brief explanation of the given instructions would strengthen the methods section.

Reviewer #4: The authors report on acute CMJ changes immediately and at 24 and 48hrs following a volleyball match in a small group of semi professional female players. The authors do not have the sample size necessary to be sub grouping the cohort into low and high playing minutes, and in fact they need to be more cautious in their overall interpretation due to even the overall sample size being low for the types of interpretations and analysis performed. The manuscript is well written and easy to read but the interpretations and outcomes need adjusting to reflect the sample size.

Specific Comments

Abstract - Methods; This study is not a longitudinal study as described in the methods it is purely an acute single data series collection. Please remove the reference to a longitudinal study design. For this study to have been longitudinal data collection would need to have been performed after multiple matches, which would have significantly improved the strength of this investigation.

Abstract - Conclusion; This conclusion does not give enough consideration to the small sample size of this study.

Methods - Design; As stated the study is not longitudinal, but I agree that yes it is repeated measures of an acute response.

Methods - Participants; A sample size power calculation needs to be provided here to justify the inclusion of only an n=12 overall and that the sub-grouping analysis was sufficient to be performed with a 2-way ANOVA.

Methods - CMJ; Can the authors please include the ICC, CV and Smallest worthwhile change scores for these variables in this cohort of athletes so the reader can understand and interpret the magnitude of change relative to this cohort of athletes. Interpreting the changes relative to ICC, CV and Smallest worthwhile change scores reported by other authors is not informative.

Methods - Statistical Analysis; As stated justify the sample size relative to this inferential statistical method or apply a different statistical approach that is less reliant on the sample size.

Results; There is data reported here of significant changes in the High minutes group at 24 and 48hrs which does not align with statements made of no change in the abstract. The abstract needs to be adjusted to reflect that no change occurred only immediately post the match.

Discussion - 2nd Paragraph; the opening sentence needs a time statement included as the match did not immediately post influence CMJ variables but there were changes observed at 24 and 48hrs.

Conclusion; Needs to be reworded to be more specific to the immediate post time point

Reviewer #5: Manuscript: Monitoring jumping ability and subjective recovery in women's volleyball: acute effects of match play on countermovement jump and well-being

Comments to authors:

The present study explores how measures of jumping ability and well-being changes due to match play and subsequent recovery in volleyball players. The paper is written concisely.

There are, however, major concerns, as listed below, along with other minor concerns (discussed in each section of the manuscript).

Major concern:

• Many general statements regarding the use and effectiveness of athlete monitoring are made in the abstract and introduction without any proper referencing (citing articles) of where these conclusions came from.

• Results: A main effect of time is shown. However, this does not imply that values are different at all time points. Please give further analysis of which time points are in fact different.

• The discussion lacks mention of the subjective measures that were not different between time points and groups. Why was this not different?

• The discussion seems to indicate different ideas about what a good time would be do to do the next training session - this needs to be clearly explained.

Other minor concerns:

Abstract:

Line 2: ‘Monitoring it helps optimize performance and prevent injuries’. I do not think this statement is necessarily valid in general and it should therefore not be part of the abstract.

Introduction:

1st paragraph:

Line 2: Remove ‘…’ after serve

Line 5: ‘To maximize performance, it is necessary to monitor athletes with the highest possible frequency, which allows to identify the relationship between load and injury risk.’ This is not true. Does ‘highest possible frequency’ imply every second, every minute, every day, or … Also, if such a general statement is going to be made, there has to be studies referenced to show that this is in fact true.

Line 9: ‘The benefits of monitoring athletes are numerous: it allows for …’. Provide references to show that this is actually true.

2nd paragraph:

Line 1: ‘In addition to being related to performance, jumping ability is a sensitive marker of fatigue, which can help identify athletes' readiness for the next competition and/or training session.’ Again, this is a statement without evidence (in the form of references) to proof that this is actually true.

Line 11: ‘Given its characteristics, the analysis of alternative metrics to traditional ones is an excellent method for monitoring athlete fatigue.’ What are the alternative vs traditional metrics mention here – please clarify.

Last line: ‘ … metrics derived from CMJ and TQR according to match loads.’ TQR and HI was initially mentioned as subjective measures, and then only TQR. Is there a reason for this selection (excluding HI)? Please explain.

Methods:

Design

Line 1: This design part shown no mention of TQR and when it was measured. Please add.

Can the authors briefly explain why PCA is specifically used. possible with refence to other studies that have done so?

Warmup

Last line: ‘To ensure optimal performance in the subsequent evaluations, the intensity of the warm-up progressively increased’ It is unclear what this means. Does the warm-up become more difficult at 24, 48, and 72 hours? How is that controlled for?

Training and competition loads quantification

Line 1: ‘To analyze the match demands, an arbitrary units measure will be used, …’. Change ‘will be used’ to ‘was used’.

Statistical Analysis

Line 5: Nowhere in the introduction or aim of the study was playing time mentioned. If you are interested in how playing time affects outcomes, it should be included in your introductory discussion.

Results:

Line 9: ‘A main effect of time was observed for …’ The fact that there was a main effect, does not show which times were different. Were all times different from all other times? If so, please state this.

Discussion:

The discussion has very little mention of the fact that TQR and HI results did not change at all. Please address this more. What have other studies found.

It appears that there are arguments for having training session at least 24-48 hours after competition (‘Following our results and the results obtained in other sports, it seems necessary to allow 24 to 48 hours between a fatiguing training or competition stimulus and the next session, if the goal is to arrive with minimal fatigue for the subsequent session.’ However, later in the discussion it appears to show that MD+1 training should be used (‘In the case of volleyball, it may be advisable to conduct an intense training session on MD+1, provided that the state of recovery allows it.’) These two ideas seem contradictory and should be explained more clearly.

**********

what does this mean?). If published, this will include your full peer review and any attached files.

If you choose “no”, your identity will remain anonymous but your review may still be made public.

**Do you want your identity to be public for this peer review?**  For information about this choice, including consent withdrawal, please see our Privacy Policy

Reviewer #1: No

Reviewer #2: No

Reviewer #3: No

Reviewer #4: **Yes:**  Dale Wilson Chapman

Reviewer #5: No

**********

[NOTE: If reviewer comments were submitted as an attachment file, they will be attached to this email and accessible via the submission site. Please log into your account, locate the manuscript record, and check for the action link "View Attachments". If this link does not appear, there are no attachment files.]

To ensure your figures meet our technical requirements, please review our figure guidelines: https://journals.plos.org/plosone/s/figures

You may also use PLOS’s free figure tool, NAAS, to help you prepare publication quality figures: https://journals.plos.org/plosone/s/figures#loc-tools-for-figure-preparation.

NAAS will assess whether your figures meet our technical requirements by comparing each figure against our figure specifications.

Attachment Submitted filename: PONE-D-25-34659_annotated.pdf

# Author comment: Author response to Decision Letter 1

**DOI:** 10.1371/journal.pone.0328664.r002

8 May 2026

Response to Reviewers

Manuscript title: Monitoring jumping ability and subjective recovery in women's volleyball: acute effects of match play on countermovement jump and well-being

Manuscript ID: PONE-D-25-34659

Dear Editor and Reviewers,

We sincerely thank you for your careful evaluation of our manuscript and for the constructive and insightful comments provided. We greatly appreciate the time and expertise dedicated to this review process.

The comments have been extremely valuable and have allowed us to substantially improve the manuscript in terms of clarity, methodological transparency, and interpretative rigor. In particular, the revised version addresses the concerns raised by improving the structure and readability of the text, clarifying the rationale for the selected variables, refining the methodological descriptions, and adopting a more cautious and precise interpretation of the findings.

Below, we provide a point-by-point response to all comments provided buy reviewers in the decision letter.

Reviewer #1

Response: We sincerely thank the reviewer for the careful and constructive evaluation of our manuscript. We fully agree that the original version required substantial revision in terms of clarity, structure, and interpretative caution.

In response to these comments, we comprehensively revised the manuscript. First, we reduced repetition and rewrote several sections of the Introduction and Discussion to improve readability, precision, and accessibility.

Second, we selected these metrics because they have been consistently described in the scientific literature as relevant indicators for assessing neuromuscular fatigue and monitoring performance-related changes in athletes. However, we acknowledge the reviewer’s important point regarding the interpretation of concentric impulse. Accordingly, we revised the Discussion to recognize that concentric impulse may reflect both force-related and time-related components, and we now interpret this variable more cautiously.

Regarding the time-related variables, we fully agree that the original interpretation was too strong given their poor reliability values. To address this concern, we added a specific paragraph in the Results section stating that movement time and time to peak power will not be further interpreted due to their low reproducibility and limited capacity to detect true changes during CMJ monitoring. Accordingly, these variables have been removed from the subsequent interpretation of the findings, and the Discussion, practical implications, and Conclusions have been revised to avoid drawing inferences from these outcomes.

Finally, we clarified the source of the data used for the reliability analyses and expanded the methodological explanation of these calculations. We also incorporated a more cautious discussion of the variability observed across post-match time points and its possible influence on reliability and sensitivity to detect meaningful effects.

We are grateful for the reviewer’s detailed annotated comments, which were instrumental in improving the manuscript substantially.

Reviewer #2

Comment:

Summary: Biomechanical variables involved in jump mechanics of semi-professional level female volleyball players were evaluated before, immediately after one day after, two days after, and three days after match play.

Comments: The authors have a good premise for assessing female volleyball players. How does the neuromuscular system recovery after a match in regards to counter-movement jump performance? There are a number of limitations which the authors acknowledge for this study. The largest limitation is the grouping of players into high match-load versus lower match load. With an N = 12, this significantly reduces the statistical power. The authors also did not make it clear in the text of the document how many players were in each group. It is also not clear how the training cycle influenced these results. The authors discuss the cycles of training, but this was not mentioned as a mitigating factor in this study. Another aspect of the study which is not mentioned is the positioning of the players. Typically, in a volleyball match the front line players jump more frequently than the other players on the court. This was not a factor addressed by this manuscript. The lack of a control group and the number of participants makes it difficult to readily evaluate the overall meaning of these data.

Response:

We sincerely thank the reviewer for the positive assessment of the study premise and for the thoughtful methodological comments.

We fully acknowledge that the small sample size is a major limitation, particularly when participants are subdivided into groups according to playing time. In the revised manuscript, we have made this limitation more explicit and have substantially moderated our interpretation of the subgroup analyses. We now clearly state that these findings should be interpreted as preliminary and context-specific rather than broadly generalizable. We have also clarified the number of participants included in each subgroup in the revised Methods/Results sections.

Regarding the influence of the training cycle, we agree that this factor required clearer treatment. We have revised the manuscript to explain more explicitly that the MD+1 training session likely influenced the post-match recovery profile and may have contributed to the changes observed at later time points, particularly at MD+2. We now discuss training load as an important contextual factor and interpret the findings accordingly.

We also agree that player position may influence match demands and jump exposure. Unfortunately, this variable was not included in the original study design and therefore could not be analyzed. We have now acknowledged this explicitly as an additional limitation in the revised Discussion, noting that positional role may affect the external and neuromuscular demands experienced during competition.

Finally, we agree that the absence of a control group and the limited number of participants restrict the strength of the conclusions. Accordingly, the revised manuscript places greater emphasis on these methodological limitations and avoids overly strong practical inferences. We are grateful for these comments, which have helped improve the transparency and overall balance of the manuscript. Nevertheless, despite these constraints, the study offers a high degree of ecological validity, as data were collected within a real-world, high-performance environment where experimental control is often not feasible. This context enhances the practical relevance of the findings, as they reflect authentic training and competition demands rather than artificially controlled conditions. Consequently, the results provide meaningful applied insights for practitioners working in similar elite settings, supporting evidence-informed decision-making while acknowledging the inherent methodological limitations.

Specific comments

Comment (Methods):

How specific are the demographic information? It seems that if height was measured in cm, then a standard deviation of 6 cm will suffice and not 6.01 cm, likewise, the standard deviations for age and mass should be to one decimal point.

Response: We thank the reviewer for this observation. The demographic data were revised to improve consistency and to avoid reporting an unnecessary degree of precision.

Comment (Methods):

If N = 12 and there was a comparison between groups – and possibly uneven numbers between groups, the statistical power would seem to be lowered. Was a power analysis conducted prior to the initiation of the study? The authors do not provide the number of participants for each group.

Response: We thank the reviewer for this important comment. No a priori power analysis was conducted prior to data collection. We acknowledge this as a limitation of the study and have now explicitly stated this in the revised manuscript. In addition, we have substantially moderated the interpretation of the subgroup analyses and inferential results accordingly. We also stated clearly in the methods section the number of participants in each group.

Comment (Methods):

An explanation of the time periods of data collection are not clear. A pre-test was performed and then two post-match tests, but the authors do not effectively communicate the durations and specific time periods of data collection as these seem to be pertinent to their study.

Response: We thank the reviewer for this comment. We revised the description of the study design and clarified all assessment time points in the Methods section and in Figure 1 to ensure consistency and transparency. The revised manuscript now clearly distinguishes pre-match, immediately post-match, and 24-, 48-, and 72-hour post-match assessments.

Comment (Results):

The authors do not refer to the data in the figures while reporting the statistics of each variable.

Response: We thank the reviewer for this observation. In the revised Results section, we improved the integration between the statistical reporting and the corresponding figures, so that the reader can more easily follow the results.

Comment (Results):

There is inconsistent reporting of the periods of testing, and it becomes very difficult to determine if these data align with their purpose.

Response: We thank the reviewer for this comment. We carefully revised the terminology and time-point labels throughout the manuscript to ensure consistency across the Abstract, Methods, Results, Discussion, Figures, and Table.

Comment (Discussion):

The limitations of this study would seem to outweigh the ramifications of the results.

Response: We appreciate this important comment. In the revised manuscript, we substantially softened the interpretation of the findings and the practical applications. We now present the results as preliminary observations from a specific ecological setting, and we explicitly acknowledge that the methodological limitations restrict the strength of the conclusions.

Reviewer #3

Comment:

The study concerns “neuromuscular and subjective recovery in female volleyball players after match play”. The topic is relevant, the work is original, and the methods are generally sound. However, several major issues limit the strength of the findings and prevent the manuscript from meeting the publication standards required by PLOS ONE.

Response: We sincerely thank the reviewer for recognizing the relevance and originality of the topic, as well as the general soundness of the methods. We also appreciate the clear identification of the major limitations. These comments were extremely helpful and led us to substantially revise the manuscript, especially regarding methodological transparency, the interpretation of the findings, and the practical conclusions.

Major concerns

Comment:

The study includes only 12 players. They are then divided into two smaller subgroups (high play time; low play time). With such low numbers, especially when subjective measures are involved, the results cannot be generalised with confidence. Individual variation will strongly influence the findings. The sample is simply too small to support the conclusions drawn.

Response: We fully agree with the reviewer that the sample size is a major limitation, particularly for subgroup analyses. In the revised manuscript, we have explicitly acknowledged this issue in the Methods, Discussion, and Conclusion sections, and we have substantially moderated the interpretation of the findings. We now present the results as exploratory and context-specific, and we avoid broad generalizations.

Comment:

Moreover, there are concerns about reproducibility – recovery is influenced by many factors like sleep, nutrition, daily routines, stress, training load, match dynamics, etc. These were not controlled or fully reported. Without this information, the study is difficult to reproduce, and the results highly depend on context.

Response: We thank the reviewer for this important observation. We agree that recovery is multifactorial and strongly influenced by contextual variables that were not fully controlled in the present study. In the revised manuscript, we acknowledged this limitation more explicitly and emphasized that the findings must be interpreted within the specific ecological context in which the data were collected. We also added discussion regarding the likely influence of contextual variables such as training load and match-related demands.

Comment:

On page 13, two key CMJ strategy variables (contraction time & time to peak power) show poor ICC reliability (ICC < 0.5). Because of this, they cannot be used confidently to support claims about strategy or neuromuscular changes. Their low reliability undermines part of the interpretation.

Response: We fully agree with the reviewer. In the revised manuscript, we substantially reduced the interpretative emphasis placed on these variables. We now explicitly state that contraction time and time to peak power showed poor reliability and, therefore, should be interpreted with caution. We also revised the Discussion and Conclusion to avoid strong claims based on these variables.

Comment:

In my opinion, also use of median split is another issue. Dividing the sample into “high” and “low” play-time groups using median split is problematic especially with only 12 study participants. It reduces statistical power and not reflects meaningful physiological groups. This approach in my opinion strongly weakens the analysis.

Response: Thank you for this comment. We acknowledge that the use of a median split in small samples can reduce statistical power and may not accurately reflect true physiological differences between groups. However, in this study the grouping based on playing time was not intended to establish distinct physiological categories, but rather to reflect a practical decision-making framework commonly used by coaching and performance staff in applied settings. In team sports such as volleyball, practitioners frequently dichotomize players (e.g., higher vs. lower match exposure) to guide load redistribution during the microcycle, particularly in MD+1 sessions. Therefore, the median split was used here as an operational proxy to reproduce this real-world approach within the analytical framework. To address this concern, we have revised the manuscript to clarify that this grouping strategy is context-driven and exploratory in nature, rather than a definitive classification of physiological status. We also explicitly acknowledge its limitations, including the potential loss of statistical sensitivity, and encourage future studies with larger samples to model playing time as a continuous variable or to apply more robust analytical approaches (e.g., mixed models) to better capture dose–response relationships.

Comment:

In volleyball practice, the paper also recommends planning high-intensity sessions the day after a match. Given the small sample, low reliability of several variables, and context-dependent measures, these conclusions should be more cautious.

Response: We fully agree. In the revised manuscript, we moderated the practical recommendations considerably. Rather than recommending high-intensity training on MD+1 in general terms, we now state that training prescription should be individualized and interpreted in light of recovery status, training context, and the cumulative effect of both match and subsequent training load.

Minor comments

Comment:

Please define all abbreviations on first use. Terms such as RPE, MDC, CI, SEM, CV, RSI and ICC should be explained clearly when they first appear.

Response: We thank the reviewer for this comment. All abbreviations were revised and are now defined at first use throughout the manuscript.

Comment:

Similarly, thoroughly define all measured variables. All CMJ-related variables should be described fully to ensure reproducibility. Similar, TQR, HI and RPE depend heavily on how athletes are instructed. A brief explanation of the given instructions would strengthen the methods section.

Response: We thank the reviewer for this valuable suggestion. The Methods section was revised to more clearly define the selected CMJ variables and to provide additional detail reg

Attachment Submitted filename: Response to Reviewers.docx

# Peer review: Decision Letter 1

**DOI:** 10.1371/journal.pone.0328664.r003

**Authors:** Mário Espada

4 Jun 2026

Dear Dr. González-García,

Thank you for submitting your manuscript to PLOS ONE. After careful consideration, we feel that it has merit but does not fully meet PLOS ONE’s publication criteria as it currently stands. Therefore, we invite you to submit a revised version of the manuscript that addresses the points raised during the review process.

Please submit your revised manuscript by Jul 19 2026 11:59PM. If you will need more time than this to complete your revisions, please reply to this message or contact the journal office at plosone@plos.org. When you're ready to submit your revision, log on to https://www.editorialmanager.com/pone/ and select the 'Submissions Needing Revision' folder to locate your manuscript file.

- A letter that responds to each point raised by the academic editor and reviewer(s). You should upload this letter as a separate file labeled 'Response to Reviewers'.
- A marked-up copy of your manuscript that highlights changes made to the original version. You should upload this as a separate file labeled 'Revised Manuscript with Track Changes'.
- An unmarked version of your revised paper without tracked changes. You should upload this as a separate file labeled 'Manuscript'.

If you would like to make changes to your financial disclosure, please include your updated statement in your cover letter. Guidelines for resubmitting your figure files are available below the reviewer comments at the end of this letter.

If applicable, we recommend that you deposit your laboratory protocols in protocols.io to enhance the reproducibility of your results. Protocols.io assigns your protocol its own identifier (DOI) so that it can be cited independently in the future. For instructions see: https://journals.plos.org/plosone/s/submission-guidelines#loc-laboratory-protocols. Additionally, PLOS ONE offers an option for publishing peer-reviewed Lab Protocol articles, which describe protocols hosted on protocols.io. Read more information on sharing protocols at https://plos.org/protocols?utm_medium=editorial-email&utm_source=authorletters&utm_campaign=protocols.

As the corresponding author, your ORCID iD is verified in the submission system and will appear in the published article. PLOS supports the use of ORCID, and we encourage all coauthors to register for an ORCID iD and use it as well. Please encourage your coauthors to verify their ORCID iD within the submission system before final acceptance, as unverified ORCID iDs will not appear in the published article. *Only*  the individual author can complete the verification step; PLOS staff *cannot*  verify ORCID iDs on behalf of authors.

We look forward to receiving your revised manuscript.

Kind regards,

Mário Espada, PhD

Academic Editor

PLOS One

Journal Requirements:

1. If the reviewer comments include a recommendation to cite specific previously published works, please review and evaluate these publications to determine whether they are relevant and should be cited. There is no requirement to cite these works unless the editor has indicated otherwise.

2. Please review your reference list to ensure that it is complete and correct. If you have cited papers that have been retracted, please include the rationale for doing so in the manuscript text, or remove these references and replace them with relevant current references. Any changes to the reference list should be mentioned in the rebuttal letter that accompanies your revised manuscript. If you need to cite a retracted article, indicate the article’s retracted status in the References list and also include a citation and full reference for the retraction notice.

Additional Editor Comments:

Dear Authors,

Congratulations on your work.

Please revise the manuscript in light of the reviewers' suggestions.

Thank you.

Best regards.

[Note: HTML markup is below. Please do not edit.]

Reviewers' comments:

Reviewer's Responses to Questions

**Comments to the Author**

Reviewer #4: (No Response)

Reviewer #6: (No Response)

Reviewer #7: All comments have been addressed

**********

2. Is the manuscript technically sound, and do the data support the conclusions?

Reviewer #4: Yes

Reviewer #6: No

Reviewer #7: Partly

**********

3. Has the statistical analysis been performed appropriately and rigorously?

Reviewer #4: Yes

Reviewer #6: Yes

Reviewer #7: No

**********

4. Have the authors made all data underlying the findings in their manuscript fully available?

The PLOS Data policy

Reviewer #4: Yes

Reviewer #6: No

Reviewer #7: Yes

**********

5. Is the manuscript presented in an intelligible fashion and written in standard English?

Reviewer #4: No

Reviewer #6: Yes

Reviewer #7: Yes

**********

Reviewer #4: The authors have made substantial changes to the manuscript in response to the first round review which has significantly improved the integrity and reproducibility. The are however some grammatical errors and the interpretation can be clarified with some adjustments. In particular the authors should rewrite any sentences using an en dash as a writing emphasis, as it is poor quality lazy writing.

Specific Comments

Ln 60-63; Reword to remove the use of the en dash

Ln 65; This is not the start of new paragraph and the word ‘assists’ should not be plural.

Ln 66; change ‘its’ to ‘of’

Ln 69; Delete ‘This assessment of’ and replace with, ‘Assessing…’

Ln 74; Insert ‘CMJ’ so the sentence reads, “….alternative CMJ metrics represents an effective approach…”

Ln 79; Delete the word ‘their’

Ln 91; Poor grammar re word, delete the words “It has” and replace with, “For example the HI has shown…”

Ln 93; poor grammar, re-word and combine these two sentences so that it reads,… “72 hours post-match, supporting the use and combination of subjective assessments….”

Ln 97; Add space after ‘sports’

Ln 239; I agree with the author’s statement but I suggest adding a further clarifying comment, to the effect of, "However match plus training loads on MD+1 does significantly impact on jump metrics at subsequent timepoints pointing towards an accumulative effect of high neuromuscular loads."

Ln 239; While understand why the authors have used the term "In contrast" here, if the suggested added statement above is included I do not believe that this is necessary and the idea flows better to the comparison to other sports.

Ln 255-263; I agree with this added interpretation however I suggest the authors consider the work of Hughes et al (2021) JSCR as one available example to support this discussion point.

Ln 269-272; I agree with this point but as suggested this factor could be introduced or at the least alluded to earlier.

Ln 274-275; Poor grammar and use of the en dash, reword, "...time, who in contrast were those exposed...MD+1, ..."

Ln 276; Suggest a wording change, delete the words ‘again showed’ and replace as, “…impulse, recorded maximal…”

Ln 281-282; Poor grammar and use of the en dash, reword, "..metrics, such as concentric impulse is enhanced, reinforcing the value of this metric for..."

Ln 290; Change the word ‘showed’ to ‘recorded’. It is not possible for a variable to have an action. Delete the word ‘which’, include a comma after the citation and change ‘suggests’ to ‘suggesting’

Ln 317; Change “This stability…’ to “The stability of response in these measures suggests…”

Ln 318; Change ‘by’ to ‘in’

Ln 319; Add (HI) after measures

Ln 326; Poor grammar and use of the en dash, reword.

Ln 348; Remove the word 'may' and make the word ‘limit’ plural.

Ln 348-349; Add a statement after this sentence, "However it is not ecologically or ethically valid in this applied setting to include a control condition."

Ln 351; Add after the word ‘addition’, ‘to this point’

Ln 352; Insert after the word ‘influenced’ and delete the word ‘both’ “..our ability to fully interpret the…”

Ln 355; Insert the following after the word “…considered,” “due to our limited sample size, however this factor in future investigations may highlight potential differences...

Reviewer #6: This study aims to analyse biomechanical CMJ variables in semi-professional female volleyball players before, immediately after and 24, 48, 72 hours following a competitive match play. Although the authors acknowledge several limitations, there is one fundamental issue that cannot be adequately resolved through limitation statements alone. the title and abstract lead the reader to expect an analysis of CMJ variations attributable to match play and subsequent recovery. However, this interpretation is problematic because the athletes performed a training session at MD+1. Consequently, comparisons between pre-match values and measurements obtained after this training session cannot be interpreted as reflecting the isolated effects of match play or recovery from competition alone. Moreover, the manuscript does not report whether athletes instructions regarding recovery-related activities, such as stretching, additional physical activity or other at-home practices. Together with the inclusion of the training session and all other limitations listed in the manuscript, these uncontrolled factors make it difficult to attribute CMJ variations specifically to match-induced fatigue and recovery.

Reviewer #7: The study attempts to quantify neuromuscular fatigue and performance in experienced women’s volleyball players via the counter movement jump (CMJ), rate of perceived exertion (RPE), total quality recovery scale (TQR), and the Hooper Index (HI). Whereas variables associated with CMJ are related to objective measures such as jump height, contraction time, or time to peak power, the other measures are somewhat subjective.

The study is timely in that a number of other researchers have shown an interest in using metrics associated with the CMJ as gauges of neuromuscular fatigue, and the author cites a number of them throughout the paper. There is especially acute interest in volleyball and in other jumping sports as CMJ related metrics can help to decouple performance measures like jump height from how an athlete moves to obtain that result. The authors, in my opinion, describe the motivation for the study quite clearly. However, the science presented is incremental, and the authors need to explain clearly what they are bringing to the table (validation of other results in volleyball, indication that such results apply to a more elite women’s group, usage of TQR+HI in conjunction with CMJ measures?). Likewise, I concur with previous reviewers regarding the grouping – while it seems to be an attempt to control for loading, it may result in under-powered statistics. Potentially using a single group and focussing on the time response could make those results clearer.

This is at least their second attempt at publication, and it’s clear that they have tried to address previous reviewers’ comments in this draft. What follows are some specific comments, some more critical than others. I’ve put an asterisk* by the important ones.

*1) Abstract, Lines 32-33: “Significant changes were observed … in players with less playing time.” Is this true? The figures at the end of the paper don’t seem to indicate that this is the case. Later RSIMod and Concentric Impulse are noted to have significant differences (lines 217-219), but Figure 3 shows no notational indication of this.

2) Line 64 – spacing. “fatigue experienced by the athlete. [space needed] In this context, the CMJ…”

3) Line 121 – If you are abbreviating match day as MD, be consistent. You have here: “...the morning of each match day, MD+1, Md+2 and MD+3.” Your second MD is mixed case.

4) Line 142 – General style standards hew to APA (but the editors may feel this is not important). Numbers less than 10 should be spelled out, unless there’s a good reason not to (for example when specifying a range). “Participants performed three attempts…”

5) Lines 150-154 – I’m not a big fan of the list presentation. I’d prefer to see these in paragraph form OR in a table, not as an unmarked list. I’ll defer to the editors again on this.

6) Line 174 – “scores of the 4 questionnaire items” → “scores of the four questionnaire items” - again this is editorial preference.

*7) Line 180 – Weir’s 2005 paper makes a point of breaking down several different ICCs in the Shrout and Fleiss framework and which is appropriate to a given study. The question is: are you looking for real consistency (ICC(3,1)) or are you looking for absolute agreement (ICC(2,1))? You should specify more clearly what you’re after from your ICCs because they will give you very different values dependent on which one you use.

8) Line 186 – “CI95%” should probably be “CI 95%”.

*9) Line 191 – “The Bonferroni correction was applied for post-hoc correction”. The Bonferroni correction is known to be overly conservative in most cases. While it reduces the chance of false positives, it also increases the chance of false negatives – often more than is warranted. Unless you have a good reason for using it (for example you didn’t do every possible post-hoc), I would go with Holm-Bonferroni or possibly Tukey to avoid overly increasing the possibility of false negatives. In short, please give a good explanation for your choice of this stat since it could affect your findings.

*10) Line 197 – The jump height ICC is suspicious in that its interval values range from 0.01-0.99 rendering the value of ICC = 0.98 nearly meaningless. In fact, the confidence interval may be more meaningful than the ICC in this case. Note that with small sample sizes, the ICC can be really unstable in that a small change in one athlete’s score could substantively change the ICC value. This suggests that the authors should be very careful interpreting this result and should not say on line 199 that it “demonstrated excellent reliability”.

11) Lines 207-219 – If ES is intended to be the Cohen d main effect size, you should indicate that this is what it is before using this abbreviation. Apologies if I missed the definition.

*12) Line 218 – You publish a concentric impulse effect size of (ES = -0.60 [-2.67 to -1.48]). Your central value is not in the interval. This needs explanation as it’s statistically implausible.

*13) Lines 239-242 – “All these findings suggest … allowing for high-load on the following sessions (MD+1)”. I disagree. Delayed onset muscle soreness (DOMS) is a real result of eccentric loading which could plausibly explain the decrease in performance of athletes after increased loading. The onset of DOMS can range from 6-24 hours and can peak between 1-3 days after the activity that caused it. I strongly suspect that a hard training day post-game will lead to longer down time for athletes later. I feel that your statement confuses the ability to train (“I don’t feel sore yet”) with best training practices. See these sources below. They may play a role in your discussion.

https://www.germanjournalsportsmedicine.com/archive/archive-2024/issue-5/update-delayed-onset-muscle-soreness-doms-muscle-biomechanics-pathophysiology-and-therapeutic-approaches

https://pmc.ncbi.nlm.nih.gov/articles/PMC11012564/

**********

what does this mean?). If published, this will include your full peer review and any attached files.

If you choose “no”, your identity will remain anonymous but your review may still be made public.

**Do you want your identity to be public for this peer review?**  For information about this choice, including consent withdrawal, please see our Privacy Policy

Reviewer #4: **Yes:**  Dale W Chapman

Reviewer #6: No

Reviewer #7: **Yes:**  Timothy A Niiler

**********

[NOTE: If reviewer comments were submitted as an attachment file, they will be attached to this email and accessible via the submission site. Please log into your account, locate the manuscript record, and check for the action link "View Attachments". If this link does not appear, there are no attachment files.]

To ensure your figures meet our technical requirements, please review our figure guidelines: https://journals.plos.org/plosone/s/figures

You may also use PLOS’s free figure tool, NAAS, to help you prepare publication quality figures: https://journals.plos.org/plosone/s/figures#loc-tools-for-figure-preparation.

NAAS will assess whether your figures meet our technical requirements by comparing each figure against our figure specifications.

# Author comment: Author response to Decision Letter 2

**DOI:** 10.1371/journal.pone.0328664.r004

3 Jul 2026

Letter of response

Manuscript: [PONE-D-25-34659R1] - [EMID:a033410873d87333]

Reviewer #4: The authors have made substantial changes to the manuscript in response to the first round review which has significantly improved the integrity and reproducibility. The are however some grammatical errors and the interpretation can be clarified with some adjustments. In particular the authors should rewrite any sentences using an en dash as a writing emphasis, as it is poor quality lazy writing.

Specific Comments

Ln 60-63; Reword to remove the use of the en dash

Authors: We thank the reviewer for this comment. The use of en dashes has been revised throughout the manuscript, and they have been replaced accordingly to ensure consistency with the journal’s style requirements.

Ln 65; This is not the start of new paragraph and the word ‘assists’ should not be plural.

Authors: We thank the reviewer for this comment. The text has been revised accordingly: the paragraph structure has been corrected, and the verb has been adjusted by removing the plural form to ensure grammatical accuracy.

Ln 66; change ‘its’ to ‘of’

Authors: We thank the reviewer for this suggestion. The wording has been corrected as recommended, replacing “its” with “of” to improve grammatical accuracy and clarity.

Ln 69; Delete ‘This assessment of’ and replace with, ‘Assessing…’

Authors: We thank the reviewer for this suggestion. The sentence has been revised as recommended by deleting “This assessment of” and replacing it with “Assessing…” to improve clarity and conciseness.

Ln 74; Insert ‘CMJ’ so the sentence reads, “….alternative CMJ metrics represents an effective approach…”

Authors: We thank the reviewer for this suggestion. The sentence has been revised accordingly by inserting “CMJ” so that it now reads: “…alternative CMJ metrics represents an effective approach…”.

Ln 79; Delete the word ‘their’

Authors: We thank the reviewer for this suggestion. The word “their” has been deleted as recommended to improve grammatical clarity.

Ln 91; Poor grammar re word, delete the words “It has” and replace with, “For example the HI has shown…”

Authors: We thank the reviewer for this indication. The sentence has been revised accordingly by deleting “It has” and replacing it with “For example, the HI has shown…” to improve grammatical accuracy and clarity.

Ln 93; poor grammar, re-word and combine these two sentences so that it reads,… “72 hours post-match, supporting the use and combination of subjective assessments….”

Authors: We thank the reviewer for this helpful comment. The sentence has been reworded and combined as recommended to improve grammatical flow and clarity, now reading: “…72 hours post-match, supporting the use and combination of subjective assessments…”.

Ln 97; Add space after ‘sports’

Authors: We thank the reviewer for this observation. The space has been added.

Ln 239; I agree with the author’s statement but I suggest adding a further clarifying comment, to the effect of, "However match plus training loads on MD+1 does significantly impact on jump metrics at subsequent timepoints pointing towards an accumulative effect of high neuromuscular loads."

We thank the reviewer for this valuable suggestion. We agree that this additional clarification strengthens the interpretation of our findings. Accordingly, we have incorporated the following statement into the Discussion:

"However, the combination of match and training loads on MD+1 appears to influence subsequent jump performance, suggesting a cumulative effect of high neuromuscular loading across the microcycle."

We appreciate this comment, which has improved the interpretation of our findings.

Authors:

Ln 239; While understand why the authors have used the term "In contrast" here, if the suggested added statement above is included I do not believe that this is necessary and the idea flows better to the comparison to other sports.

We appreciate this suggestion. Following the inclusion of the additional explanatory statement above, we have removed the expression "In contrast" to improve the logical flow and readability of the paragraph.

Authors:

Ln 255-263; I agree with this added interpretation however I suggest the authors consider the work of Hughes et al (2021) JSCR as one available example to support this discussion point.

We thank the reviewer for this excellent suggestion. The study by Hughes et al. (2021) has now been incorporated into the Discussion to further support our interpretation regarding the cumulative neuromuscular effects observed following competition and subsequent training exposure. We believe that the inclusion of this reference strengthens the scientific context of our findings.

Authors:

Ln 269-272; I agree with this point but as suggested this factor could be introduced or at the least alluded to earlier.

Authors: We appreciate this observation. We agree that introducing this concept earlier improves the coherence and progression of the Discussion. Accordingly, we have incorporated a statement in the preceding paragraph acknowledging that the cumulative neuromuscular stimulus differed between subgroups due to their different exposure to match play and subsequent training. We believe this addition provides a clearer framework for interpreting the observed CMJ responses throughout the microcycle.

Ln 274-275; Poor grammar and use of the en dash, reword, "...time, who in contrast were those exposed...MD+1, ..."

Authors: We thank the reviewer for this helpful correction. The sentence has been reworded to improve grammatical accuracy and clarity, and the en dash has been removed. It now reads: “…time, who in contrast were those exposed to MD+1…”.

Ln 276; Suggest a wording change, delete the words ‘again showed’ and replace as, “…impulse, recorded maximal…”

Authors: We thank the reviewer for this helpful suggestion. The sentence has been revised as recommended by deleting “again showed” and replacing it with “recorded maximal…”, improving both clarity and conciseness.

Ln 281-282; Poor grammar and use of the en dash, reword, "..metrics, such as concentric impulse is enhanced, reinforcing the value of this metric for..."

Authors: We thank the reviewer for this helpful suggestion. The sentence has been reworded to improve grammatical structure and clarity, and the en dash has been removed. It now reads: “…metrics, such as concentric impulse is enhanced, reinforcing the value of this metric for…”.

Ln 290; Change the word ‘showed’ to ‘recorded’. It is not possible for a variable to have an action. Delete the word ‘which’, include a comma after the citation and change ‘suggests’ to ‘suggesting’

Authors: We thank the reviewer for this helpful observation. The sentence has been revised as recommended: “showed” has been replaced with “recorded,” “which” has been deleted, a comma has been added after the citation, and “suggests” has been changed to “suggesting” to improve grammatical accuracy and flow.

Ln 317; Change “This stability…’ to “The stability of response in these measures suggests…”

Authors: We thank the reviewer for this helpful suggestion. The sentence has been revised as recommended by changing “This stability…” to “The stability of response in these measures suggests…” to improve clarity and grammatical flow.

Ln 318; Change ‘by’ to ‘in’

Authors: We thank the reviewer for this helpful correction. The preposition has been amended as suggested, replacing “by” with “in” to improve grammatical accuracy.

Ln 319; Add (HI) after measures

Authors: We thank the reviewer for this helpful suggestion. The abbreviation (HI) has been added after “measures” as recommended to improve clarity and ensure consistency in terminology.

Ln 326; Poor grammar and use of the en dash, reword.

Authors: We thank the reviewer for this helpful correction. The sentence has been reworded to improve grammatical accuracy, and the en dashes have been removed and replaced with commas as appropriate to ensure consistency and clarity throughout the manuscript.

Ln 348; Remove the word 'may' and make the word ‘limit’ plural.

Authors: We thank the reviewer for this helpful suggestion. The sentence has been revised by removing “may” and changing “limit” to the plural form “limits” to improve grammatical accuracy and clarity

Ln 348-349; Add a statement after this sentence, "However it is not ecologically or ethically valid in this applied setting to include a control condition."

Authors: We thank the reviewer for this insightful suggestion. The sentence has been updated accordingly, and a clarifying statement has been added as recommended: “However, it is not ecologically or ethically valid in this applied setting to include a control condition.” We appreciate this valuable comment, which has strengthened the interpretation of the study limitations.

Ln 351; Add after the word ‘addition’, ‘to this point’

Authors: We thank the reviewer for this helpful suggestion. The text has been revised by adding “to this point” after “addition” to improve clarity and flow.

Ln 352; Insert after the word ‘influenced’ and delete the word ‘both’ “..our ability to fully interpret the…”

Authors: We thank the reviewer for this helpful suggestion. The sentence has been revised accordingly by inserting “our ability to fully interpret the…” after “influenced” and deleting “both” to improve grammatical accuracy and clarity.

Ln 355; Insert the following after the word “…considered,” “due to our limited sample size, however this factor in future investigations may highlight potential differences...

Authors: We thank the reviewer for this helpful suggestion. The sentence has been revised as recommended by inserting the following text after “considered,”: “due to our limited sample size, however this factor in future investigations may highlight potential differences…”, in order to improve clarity and strengthen the discussion of limitations and future directions.

Reviewer #6: This study aims to analyse biomechanical CMJ variables in semi-professional female volleyball players before, immediately after and 24, 48, 72 hours following a competitive match play. Although the authors acknowledge several limitations, there is one fundamental issue that cannot be adequately resolved through limitation statements alone. the title and abstract lead the reader to expect an analysis of CMJ variations attributable to match play and subsequent recovery. However, this interpretation is problematic because the athletes performed a training session at MD+1. Consequently, comparisons between pre-match values and measurements obtained after this training session cannot be interpreted as reflecting the isolated effects of match play or recovery from competition alone. Moreover, the manuscript does not report whether athletes instructions regarding recovery-related activities, such as stretching, additional physical activity or other at-home practices. Together with the inclusion of the training session and all other limitations listed in the manuscript, these uncontrolled factors make it difficult to attribute CMJ variations specifically to match-induced fatigue and recovery.

Authors: We thank the reviewer for this thoughtful and constructive comment. We agree that the inclusion of the MD+1 training session, together with the inability to control individual recovery-related behaviours, prevents attributing the observed CMJ responses exclusively to match play or recovery from competition.

Our intention was not to isolate the physiological effects of match play, but rather to describe the neuromuscular responses occurring throughout a real competitive microcycle under ecological conditions. To better reflect this perspective, we have revised the title, abstract, objectives, discussion, and conclusions to avoid implying causal interpretations related solely to match-induced fatigue or recovery. In addition, we have expanded the limitations section to explicitly acknowledge the potential influence of both the scheduled training session and uncontrolled recovery behaviours on the observed responses.

Now it reads: An important consideration is that the observed changes cannot be attributed exclusively to match play, as players also completed a scheduled training session on MD+1 and individual recovery-related behaviours outside the team environment (e.g., stretching, additional physical activity, or other recovery strategies) were not controlled. Consequently, the present findings should be interpreted as the neuromuscular responses occurring throughout a real competitive microcycle rather than as isolated effects of competition or recovery”

We sincerely thank the reviewer for this valuable comment, which has improved the transparency and interpretation of our study.

Reviewer #7: The study attempts to quantify neuromuscular fatigue and performance in experienced women’s volleyball players via the counter movement jump (CMJ), rate of perceived exertion (RPE), total quality recovery scale (TQR), and the Hooper Index (HI). Whereas variables associated with CMJ are related to objective measures such as jump height, contraction time, or time to peak power, the other measures are somewhat subjective.

The study is timely in that a number of other researchers have shown an interest in using metrics associated with the CMJ as gauges of neuromuscular fatigue, and the author cites a number of them throughout the paper. There is especially acute interest in volleyball and in other jumping sports as CMJ related metrics can help to decouple performance measures like jump height from how an athlete moves to obtain that result. The authors, in my opinion, describe the motivation for the study quite clearly. However, the science presented is incremental, and the authors need to explain clearly what they are bringing to the table (validation of other results in volleyball, indication that such results apply to a more elite women’s group, usage of TQR+HI in conjunction with CMJ measures?). Likewise, I concur with previous reviewers regarding the grouping – while it seems to be an attempt to control for loading, it may result in under-powered statistics. Potentially using a single group and focussing on the time response could make those results clearer.

This is at least their second attempt at publication, and it’s clear that they have tried to address previous reviewers’ comments in this draft. What follows are some specific comments, some more critical than others. I’ve put an asterisk* by the important ones.

*1) Abstract, Lines 32-33: “Significant changes were observed … in players with less playing time.” Is this true? The figures at the end of the paper don’t seem to indicate that this is the case. Later RSIMod and Concentric Impulse are noted to have significant differences (lines 217-219), but Figure 3 shows no notational indication of this.

Authors: We thank the reviewer for this important observation. We have updated the corresponding figure to ensure consistency with the statistical results presented in the manuscript. The figures now accurately reflect the significant differences reported in the abstract.

2) Line 64 – spacing. “fatigue experienced by the athlete. [space needed] In this context, the CMJ…”

Authors: Thanks to the reviewer for this observation. We have added the needed space.

3) Line 121 – If you are abbreviating match day as MD, be consistent. You have here: “...the morning of each match day, MD+1, Md+2 and MD+3.” Your second MD is mixed case.

Authors: We thank the reviewer for identifying this inconsistency. The abbreviation has now been corrected throughout the manuscript, and all references to match day are consistently reported in uppercase format (MD, MD+1, MD+2, and MD+3). We appreciate this observation, which has improved the consistency of the manuscript.

4) Line 142 – General style standards hew to APA (but the editors may feel this is not important). Numbers less than 10 should be spelled out, unless there’s a good reason not to (for example when specifying a range). “Participants performed three attempts…”

Authors: We thank the reviewer for this helpful observation. In accordance with APA style recommendations, numbers lower than ten have been revised from numerals to words where appropriate. Specifically,

Attachment Submitted filename: Letter of respose.docx

# Peer review: Decision Letter 2

**DOI:** 10.1371/journal.pone.0328664.r005

**Authors:** Mário Espada

22 Jul 2026

Dear Dr. González-García,

Thank you for submitting your manuscript to PLOS ONE. After careful consideration, we feel that it has merit but does not fully meet PLOS ONE’s publication criteria as it currently stands. Therefore, we invite you to submit a revised version of the manuscript that addresses the points raised during the review process.

Please submit your revised manuscript by Sep 05 2026 11:59PM. If you will need more time than this to complete your revisions, please reply to this message or contact the journal office at plosone@plos.org. When you're ready to submit your revision, log on to https://www.editorialmanager.com/pone/ and select the 'Submissions Needing Revision' folder to locate your manuscript file.

- A letter that responds to each point raised by the academic editor and reviewer(s). You should upload this letter as a separate file labeled 'Response to Reviewers'.
- A marked-up copy of your manuscript that highlights changes made to the original version. You should upload this as a separate file labeled 'Revised Manuscript with Track Changes'.
- An unmarked version of your revised paper without tracked changes. You should upload this as a separate file labeled 'Manuscript'.

If you would like to make changes to your financial disclosure, please include your updated statement in your cover letter. Guidelines for resubmitting your figure files are available below the reviewer comments at the end of this letter.

If applicable, we recommend that you deposit your laboratory protocols in protocols.io to enhance the reproducibility of your results. Protocols.io assigns your protocol its own identifier (DOI) so that it can be cited independently in the future. For instructions see: https://journals.plos.org/plosone/s/submission-guidelines#loc-laboratory-protocols. Additionally, PLOS ONE offers an option for publishing peer-reviewed Lab Protocol articles, which describe protocols hosted on protocols.io. Read more information on sharing protocols at https://plos.org/protocols?utm_medium=editorial-email&utm_source=authorletters&utm_campaign=protocols.

As the corresponding author, your ORCID iD is verified in the submission system and will appear in the published article. PLOS supports the use of ORCID, and we encourage all coauthors to register for an ORCID iD and use it as well. Please encourage your coauthors to verify their ORCID iD within the submission system before final acceptance, as unverified ORCID iDs will not appear in the published article. *Only*  the individual author can complete the verification step; PLOS staff *cannot*  verify ORCID iDs on behalf of authors.

We look forward to receiving your revised manuscript.

Kind regards,

Mário Espada, PhD

Academic Editor

PLOS One

Journal Requirements:

If the reviewer comments include a recommendation to cite specific previously published works, please review and evaluate these publications to determine whether they are relevant and should be cited. There is no requirement to cite these works unless the editor has indicated otherwise.

Please review your reference list to ensure that it is complete and correct. If you have cited papers that have been retracted, please include the rationale for doing so in the manuscript text, or remove these references and replace them with relevant current references. Any changes to the reference list should be mentioned in the rebuttal letter that accompanies your revised manuscript. If you need to cite a retracted article, indicate the article’s retracted status in the References list and also include a citation and full reference for the retraction notice.

Additional Editor Comments (if provided):

Dear Authors,

Congratulations on your work.

Please revise the manuscript in light of the reviewers' minor suggestions.

Thank you.

Best regards.

[Note: HTML markup is below. Please do not edit.]

Reviewers' comments:

Reviewer's Responses to Questions

**Comments to the Author**

Reviewer #4: (No Response)

Reviewer #8: (No Response)

**********

2. Is the manuscript technically sound, and do the data support the conclusions?

Reviewer #4: Yes

Reviewer #8: Yes

**********

3. Has the statistical analysis been performed appropriately and rigorously?

Reviewer #4: Yes

Reviewer #8: Yes

**********

4. Have the authors made all data underlying the findings in their manuscript fully available?

The PLOS Data policy

Reviewer #4: Yes

Reviewer #8: Yes

**********

5. Is the manuscript presented in an intelligible fashion and written in standard English?

Reviewer #4: Yes

Reviewer #8: Yes

**********

Reviewer #4: The authors have made substantial changes to the manuscript following the first review round, however there are some discrepancies between the reported changes and actual manuscript changes. For example this change in relation to this reviewer's comment is not made in the manuscript;

Authors:

Ln 255-263; I agree with this added interpretation however I suggest the authors consider the work of Hughes et al (2021) JSCR as one available example to support this discussion point.

We thank the reviewer for this excellent suggestion. The study by Hughes et al. (2021) has now been incorporated into the Discussion to further support our interpretation regarding the cumulative neuromuscular effects observed following competition and subsequent training exposure. We believe that the inclusion of this reference strengthens the scientific context of our findings.

As the authors have substantially re-worked their discussion to focus on the magnitude of change identified by the MDC, the change associated with this comment may have been deleted. However, even in the updated discussion there remains discussion on prolonged neuromuscular fatigue detection (e.g. Ln 271-273; Ln 290-292; Ln 334-337) that are not supported by evidence. The work cited of Bishop et al 2023 is a general review text and there are better primary evidence texts available which should be cited. The authors are also requested to ensure there is consistency and accuracy between the reported author responses and the revised manuscript.

Reviewer #8: This manuscript investigates the temporal patterns of neuromuscular fatigue and subjective recovery in semi-professional female volleyball players across a competitive microcycle. The authors employed countermovement jump (CMJ) metrics and subjective assessments—specifically the Total Quality Recovery (TQR) scale and the Hooper Index (HI)—to evaluate twelve athletes at multiple time points: pre-match, immediately post-match, and at 24, 48, and 72 hours following competition.

The primary findings indicate that competition did not induce immediate neuromuscular impairment; however, significant declines in jumping performance, characterized by reductions in jump height, concentric impulse, and the modified reactive strength index (RSImod), emerged 48 hours post-match. The authors interpret this delayed response as the cumulative effect of both match-induced fatigue and the subsequent training loads performed within the microcycle. Notably, subjective recovery measures remained relatively stable and did not significantly mirror these objective neuromuscular changes, suggesting a potential dissociation between perceived and actual physical readiness.

Overall, this study provides practical insights into the application of force-time metrics in an ecological sporting environment. It concludes that concentric impulse is a particularly sensitive indicator for monitoring fatigue and emphasizes the necessity of a holistic monitoring approach to individualize training prescriptions and optimize recovery in women's volleyball.

Overall Evaluation

Overall, the authors have successfully addressed the previous reviewers' comments, and the manuscript has become substantially more robust. However, I believe the following minor points should be addressed to further improve the quality of the final manuscript.

Specific Comments

1. Consistency in Decimal Places (L122, Table 1, etc.)

Inconsistencies in the number of decimal places were observed in several locations, such as L122 and Table 1. Please ensure that these are standardized throughout the manuscript, taking into account the appropriate number of significant figures for each metric.

2. Clarification of Jump Height ICC Confidence Interval (L194)

In L194, is the reported 95% CI for the Jump height ICC (0.01–0.99) indeed correct? Given that the ICC is 0.98, this interval appears unusually wide. Please double-check this value.

3. Reporting Effect Sizes for ANOVA (L204–205)

For the ANOVA results presented in L204–205, it is recommended to include the effect size (e.g., partial eta squared, ηp2) to provide a more complete statistical picture.

4. Wording for Significant Differences (L209)

In L209, since you are describing a statistically significant difference, the word "significant" should be added before "reductions" (e.g., "...significant reductions were observed...") to ensure clarity.

5. Notation of Effect Sizes in the Results Section (L210, etc.)

In the Results section (e.g., L210), it is unconventional to use the generic abbreviation "ES." Since you have calculated Cohen’s d, please use "d = XX" or "Cohen’s d = XX" for standard reporting.

6. Clarification of "each" (L252)

In L252, the term "each" in the phrase "the duration of each point" is somewhat ambiguous. Please clarify what this refers to (e.g., "each rally" or "each game point") to improve reader understanding.

7. Consistent Use of Abbreviations (L263)

In L263, since the full term "minimal detectable change" has already been defined earlier in the manuscript, you should use the abbreviation "MDC" only.

8. Spacing and Formatting (L308, etc.)

There are several instances of extra or missing spaces throughout the manuscript, including L308. Please conduct a thorough review to ensure consistent formatting.

9. Enhancement of Figure Captions (L505–506)

The captions for Figures 1 and 2 (L505–506) are currently too brief, making it difficult for the reader to interpret the data without referring back to the text. Please provide more descriptive and detailed captions for these figures.

**********

what does this mean?). If published, this will include your full peer review and any attached files.

If you choose “no”, your identity will remain anonymous but your review may still be made public.

**Do you want your identity to be public for this peer review?**  For information about this choice, including consent withdrawal, please see our Privacy Policy

Reviewer #4: **Yes:**  Dale W Chapman

Reviewer #8: **Yes:**  Yoichiro Sato

**********

[NOTE: If reviewer comments were submitted as an attachment file, they will be attached to this email and accessible via the submission site. Please log into your account, locate the manuscript record, and check for the action link "View Attachments". If this link does not appear, there are no attachment files.]

To ensure your figures meet our technical requirements, please review our figure guidelines: https://journals.plos.org/plosone/s/figures

You may also use PLOS’s free figure tool, NAAS, to help you prepare publication quality figures: https://journals.plos.org/plosone/s/figures#loc-tools-for-figure-preparation.

NAAS will assess whether your figures meet our technical requirements by comparing each figure against our figure specifications.

Attachment Submitted filename: ReviewAttachment.pdf

# Author comment: Author response to Decision Letter 3

**DOI:** 10.1371/journal.pone.0328664.r006

3 Aug 2026

Response to Reviewers

Monitoring CMJ-Derived Metrics and Subjective Recovery Across a Competitive Microcycle in Semi-Professional Women's Volleyball

Dear Editor and Reviewers,

We thank the Editor and the Reviewers for their careful assessment of the revised manuscript. We have addressed all remaining comments and conducted a final consistency and formatting check. The revisions are intentionally focused and do not alter the study design, analyses, principal findings, or overall conclusions. Our point-by-point responses are provided below. Reviewer comments are reproduced in bold, followed by our responses and the corresponding changes made in the manuscript.

Reviewer #4

The authors have made substantial changes to the manuscript following the first review round; however, there are discrepancies between the reported changes and the actual manuscript changes. In particular, the previously stated inclusion of Hughes et al. (2021) was not present. The remaining discussion of delayed or prolonged neuromuscular fatigue should also be supported by primary evidence rather than relying mainly on the general review by Bishop et al. (2023). The authors should ensure consistency between the response letter and the revised manuscript.

Response: We agree with the Reviewer and apologize for the discrepancy between our previous response and the revised manuscript. During the substantial restructuring of the Discussion, the sentence containing the citation to Hughes et al. was inadvertently removed. We have now corrected this inconsistency. The study by Hughes et al. has been incorporated into the Discussion as primary experimental evidence showing that fatigue-related changes can be detected across the CMJ force-time curve and may not be fully represented by jump height alone. In addition, we revised several statements concerning delayed neuromuscular fatigue so that they are framed more cautiously and linked directly to the evidence available from the present data and primary studies.

Reviewer #8

1. Consistency in Decimal Places (L122, Table 1, etc.). Inconsistencies in the number of decimal places were observed. Please standardize these throughout the manuscript, taking into account the appropriate number of significant figures for each metric.

Response: We agree. The numerical values in Table 1 have been standardized and rounded to zero decimal places to ensure consistent reporting within the table. inferential statistics and reliability coefficients to two decimal places where appropriate, and p values to three decimal places, except when p < 0.001.

2. Clarification of Jump Height ICC Confidence Interval (L194). Is the reported 95% CI for the jump height ICC (0.01–0.99) correct? Given that the ICC is 0.98, this interval appears unusually wide. Please double-check this value.

Response: We thank the Reviewer for identifying this issue. The lower confidence limit was a transcription error. We rechecked the reliability output and corrected the confidence interval in the manuscript.

3. Reporting Effect Sizes for ANOVA (L204–205). For the ANOVA results, include an effect size such as partial eta squared (ηp²).

Response: We agree and have added partial eta squared values for the significant main effects of time.

4. Wording for Significant Differences (L209). Add the word “significant” before “reductions.”

Response: We agree and have revised the sentence accordingly.

5. Notation of Effect Sizes in the Results Section (L210, etc.). Since Cohen’s d was calculated, use “d = XX” rather than the generic abbreviation “ES.”

Response: We agree. The generic abbreviation “ES” has been replaced by Cohen’s d notation throughout the Results and in the relevant parts of the Discussion.

6. Clarification of “each” (L252). The phrase “the duration of each point” is ambiguous.

Response: We agree and have clarified that the term refers to each rally.

7. Consistent Use of Abbreviations (L263). Since minimal detectable change has already been defined, use “MDC” only.

Response: We agree and have removed the repeated definition.

8. Spacing and Formatting (L308, etc.). Conduct a thorough formatting review.

Response: We agree. We conducted a complete proofread and corrected missing or extra spaces, punctuation, unit formatting, and typographical errors throughout the manuscript. PENDIETEEEE

9. Enhancement of Figure Captions (L505–506). The captions for Figures 1 and 2 are too brief. Please provide more descriptive captions.

Response: We agree. All figure captions have been expanded so that the figures can be interpreted independently from the main text. The revised captions define the time points, groups, symbols, summary statistics, error bars, and significance annotations.

Attachment Submitted filename: response_reviewers.docx

# Peer review: Decision Letter 3

**DOI:** 10.1371/journal.pone.0328664.r007

**Authors:** Mário Espada

17 Aug 2026

<p>Monitoring CMJ-Derived Metrics and Subjective Recovery Across a Competitive Microcycle in Semi-Professional Women's Volleyball

PONE-D-25-34659R3

Dear Dr. Jaime González-García,

We’re pleased to inform you that your manuscript has been judged scientifically suitable for publication and will be formally accepted for publication once it meets all outstanding technical requirements.

Within one week, you’ll receive an e-mail detailing the required amendments. When these have been addressed, you’ll receive a formal acceptance letter and your manuscript will be scheduled for publication.

An invoice will be generated when your article is formally accepted. Please note, if your institution has a publishing partnership with PLOS and your article meets the relevant criteria, all or part of your publication costs will be covered. Please make sure your user information is up-to-date by logging into Editorial Manager at Editorial Manager® and clicking the ‘Update My Information' link at the top of the page. For questions related to billing, please contact billing support.

If your institution or institutions have a press office, please notify them about your upcoming paper to help maximize its impact. If they’ll be preparing press materials, please inform our press team as soon as possible -- no later than 48 hours after receiving the formal acceptance. Your manuscript will remain under strict press embargo until 2 pm Eastern Time on the date of publication. For more information, please contact onepress@plos.org.

Kind regards,

Mário Espada, PhD

Academic Editor

PLOS One

# Editor report: Acceptance letter

**DOI:** 10.1371/journal.pone.0328664.r008

**Authors:** Mário Espada

PONE-D-25-34659R3

PLOS One

Dear Dr. González-García,

I'm pleased to inform you that your manuscript has been deemed suitable for publication in PLOS One. Congratulations! Your manuscript is now being handed over to our production team.

At this stage, our production department will prepare your paper for publication. This includes ensuring the following:

* All references, tables, and figures are properly cited

* All relevant supporting information is included in the manuscript submission,

* There are no issues that prevent the paper from being properly typeset

You will receive further instructions from the production team, including instructions on how to review your proof when it is ready. Please keep in mind that we are working through a large volume of accepted articles, so please give us a few days to review your paper and let you know the next and final steps.

Lastly, if your institution or institutions have a press office, please let them know about your upcoming paper now to help maximize its impact. If they'll be preparing press materials, please inform our press team within the next 48 hours. Your manuscript will remain under strict press embargo until 2 pm Eastern Time on the date of publication. For more information, please contact onepress@plos.org.

You will receive an invoice from PLOS for your publication fee after your manuscript has reached the completed accept phase. If you receive an email requesting payment before acceptance or for any other service, this may be a phishing scheme. Learn how to identify phishing emails and protect your accounts at https://explore.plos.org/phishing.

If we can help with anything else, please email us at customercare@plos.org.

Thank you for submitting your work to PLOS One and supporting open access.

Kind regards,

PLOS One Editorial Office Staff

on behalf of

Dr. Mário Espada

Academic Editor

PLOS One
